# Spherically symmetric teleparallel geometries

**DOI:** 10.1140/epjc/s10052-024-12629-5

**Published:** 2024-03-29

**Authors:** A. A. Coley, A. Landry, R. J. van den Hoogen, D. D. McNutt

**Affiliations:** 1 Department of Mathematics and Statistics, Dalhousie University, Halifax, NS B3H 3J5 Canada; 2 Department of Mathematics and Statistics, St. Francis Xavier University, Antigonish, NS B2G 2W5 Canada; 3 Centrum Fizyki Teoretycznej, Polska Akademia Nauk, Al. Lotników 32/46, 02-668 Warsaw, Poland

## Abstract

We are interested in the development of spherically symmetric geometries in *F*(*T*) teleparallel gravity which are of physical importance. We first express the general forms for the spherically symmetric frame and the zero curvature, metric compatible, spin connection. We then analyse the antisymmetric field equations (the solutions of which split into two cases, which we subsequently consider separately), and derive and analyse the resulting symmetric field equations. In order to further study the applications of spherically symmetric teleparallel models, we study 3 subcases in which there is an additional affine symmetry so that the resulting field equations reduce to a system of ordinary differential equations. First, we study static spherical symmetric geometries and solve the antisymmetric field equations and subsequently derive the full set of symmetric field equations. In particular, we investigate vacuum spacetimes and obtain a number of new solutions. Second, we consider an additional affine frame symmetry in order to expand the affine frame symmetry group to that of a spatially homogeneous Kantowski–Sachs geometry. Third, we study the special case of spherical symmetry with an additional fourth similarity affine vector.

## Introduction

In Einstein’s General theory of Relativity (GR), the spherically symmetric vacuum solution, the Schwarzschild solution, is likely the most recognized solution in all of GR. The Schwarzschild solution, one of the first solutions to the Einstein field equations (FEs), provides an excellent theoretical model for describing gravitational effects in vacuum around a non-rotating spherical mass. Within our solar system, the Schwarzschild solution provides a mechanism to explain most of the perihelion precession of Mercury that could not be ascribed to Newtonian mechanics alone. This explanation provided one of the early successful tests of GR. The importance of the Schwarzschild solution in GR cannot be understated. Indeed, Birkhoff’s theorem states that the Schwarzschild solution is the only spherically symmetric and asymptotically flat solution to the Einstein FEs. Furthermore, these Schwarzschild solutions can give rise to black hole solutions in which there is a horizon in which light ray trajectories which enter the horizon, or initially start inside the horizon, cannot escape and will approach a singularity.

Spherically symmetric solutions in any gravitational theory are clearly of the utmost importance. Most astrophysical objects are spherically symmetric to some degree, and therefore any theory of gravity should have well described spherically symmetric solutions developed so that the theory can be tested against these astrophysical observations. In this paper, a general class of spherically symmetric geometries is developed in an effort to find spherically symmetric solutions in an alternative theory of gravity to GR.

Teleparallel gravity (see [1, 2] and references within for comprehensive reviews) is an alternative to GR theory of gravity. In GR the geometry is characterized by the curvature of the Levi–Civita connection which is a function of the metric. In teleparallel gravity, the geometrical quantity of interest is the torsion which is computed from the frame (or co-frame) and a zero curvature, metric compatible, spin connection. While the theories appear very different, interestingly, the Teleparallel Equivalent to General Relativity (TEGR) is a subclass of teleparallel gravitational theories that is dynamically equivalent to GR [3]. These TEGR theories are based on a scalar, *T*, called the torsion scalar, constructed from a particular linear combination of various scalar invariants of the torsion tensor [3]. An important generalization of TEGR is *F*(*T*) teleparallel gravity, where *F* is an arbitrary twice differentiable function of the torsion scalar [1, 2, 4–8].

When the geometrical framework describing *F*(*T*) teleparallel gravity is defined in a gauge invariant manner as a geometry with a connection having zero curvature which is also compatible with the metric (that is, the non-metricity is also zero), the resulting FEs derived from such a theory are fully Lorentz covariant [7, 8]. Due to the Lorentz invariance, there will exist a frame/spin connection pair in which the spin connection identically vanishes. In this very special class of frame/spin connection pairs, the frame is called a *proper frame*. This is also known as the Weitzenbock gauge. Any teleparallel theory of gravity that restricts itself to only the proper frame is not locally Lorentz invariant. To have a locally Lorentz invariant teleparallel theory of gravity necessarily requires that we do not restrict ourselves to proper frames, and therefore the spin connection may be non-zero in these other frames [3, 8, 9]. For example, if one was to use spherical coordinates to describe a vacuum Minkowski geometry and then assume a diagonal form for the frame and a trivial spin connection, one quickly concludes that something is amiss, as the torsion would be non-nero. It is important to carefully choose a frame/spin connection pair that is in alignment with the symmetry conditions imposed.

Therefore to construct gravitational models in teleparallel gravity, one requires the development of suitable ansatzes for the frame (or co-frame) and the spin connection that respect the assumed symmetries of the situation. Since the geometrical framework is no longer that of a Riemannian geometry in which the tools to study symmetries have been well developed, one must adapt and generalize these tools to the arena of Riemann–Cartan geometries, and in our case restrict them to teleparallel geometries. The development of these tools to study symmetries in Riemann–Cartan geometries and/or teleparallel geometries is an active area of current research [10–16]. Indeed the development of a more robust understanding of symmetries in teleparallel geometries is the first step in the development physical gravitational models in teleparallel gravity. Refs. [10, 16] are equivalent. However, [16] avoids the problems in representation theory that is necessary in [10] for their approach, which can be difficult to solve for larger groups.

In this paper we shall present the frame/spin connection pair that describes the most general spherically symmetric teleparallel geometries. We then employ this frame/spin connection pair in the *F*(*T*) teleparallel gravity FEs. With the assumption of additional symmetries the FEs reduce to a system of ordinary differential equations (ODEs) suitable for further study. We study 3 such cases in which there is an additional affine symmetry. First, we investigate the FEs assuming the geometry is static and determine solutions in vacuum. We then study spatially homogeneous Kankowski–Sachs geometries. Further, with the assumption of a non-trivial similarity condition, the FEs again reduce to a system of ODEs which we analyze.

We will follow the notation of [16] and denote the coordinate indices by $\mu , \nu , \ldots$ and the tangent space indices by $a,b,\ldots$. Unless otherwise indicated the spacetime coordinates will be $x^\mu$. Round brackets surrounding indices represents symmetrization, while square brackets represents anti-symmetrization. Any underlined index is not included in the symmetrization. The orthonormal frame fields are denoted as ${\textbf{h}}_a$ and the dual coframe one-forms are ${\textbf{h}}^a$. The vierbein components are $h_a^{~\mu }$ or $h^a_{~\mu }$. The spacetime metric will be denoted as $g_{\mu \nu }$ and since we have an orthonormal frame the tangent space metric is $\eta _{ab}=Diag[-1,1,1,1]$. To denote a local Lorentz transformation leaving $\eta _{ab}$ unchanged, we write $\Lambda _a^{~b}(x^\mu )$ and express the inverse as $\Lambda _b^{~a}=(\Lambda ^{-1})^a_{~b}$. The spin connection one-form ${{{\varvec{\omega }}}}^a_{~b}$, is designated by ${{{\varvec{\omega }}}}^a_{~b} = \omega ^a_{~bc} {\textbf{h}}^c$. The spin connection one-form ${{{\varvec{\omega }}}}^a_{~b}$ defines a covariant differentiation process on a tensor $T^a_{~~b}$ as $\nabla _c\,T^a_{~~b}=h_c\,\left( T^a_{~~b}\right) +\omega ^a_{~ec}\,T^e_{~~b}-\omega ^e_{~bc}\,T^a_{~~e}=T^a_{~~b;c}$. The Torsion tensor will be defined as $T^a_{~~bc}=2h_b^{~\nu }\,h_c^{~\mu }\left( h^d_{~[\nu ,\mu ]}-\omega ^a_{~d[\mu } h^d_{~\nu ]}\right)$.

### Review of the literature

Some simple spherically symmetric teleparallel solutions, and especially static and vacuum solutions, were studied in [1] (also see references within). For example, the Birkhoff theorem does not generally hold in *F*(*T*) theories. In particular, a perturbed solution around Minkowski spacetime was first used to put solar system constraints on the parameters in *F*(*T*) gravity [17, 18].

Cosmological models in flat ($k = 0$) teleparallel Robertson–Walker (TRW) models have been studied in [1] (also see references within). For example, simple power-law scale factor solutions in particular *F*(*T*) models (using a priori ansatz such as a polynomial function) were investigated. In particular, reconstruction methods have been explored extensively in the literature (in which the function *F*(*T*) is reconstructed from the underlying assumptions of the models). Dynamical systems methods [19] (e.g., fixed point and stability analysis) in flat TRW models have been widely utilized [1] (also see [20], and [14, 21]), which includes the study of the stability of the de Sitter fixed point. In future work we will be interested in further studying the curved $k \ne 0$ TRW models.

Almost all astrophysical solutions found within *F*(*T*) theories have been restricted to the static case [1]. Static spherically symmetric solutions have been reviewed in [1], and some more recent references are noted below. Here we are only interested in *exact* solutions in non-trivial *F*(*T*) theories (i.e., $F(T) \ne T + \Lambda$ and $T \ne$ const.) that are physical (e.g., real valued tetrads). We note that many of the solutions presented in the literature are incorrect.

Finding exact vacuum static spherically symmetric *F*(*T*) teleparallel gravity solutions is an open problem, and there are no known exact vacuum black hole solutions. Indeed, the only exact non-trivial vacuum solution known to the authors in *F*(*T*) teleparallel gravity is the non-black hole power law solution presented in [22].

There are a number of special static spherically symmetric (anisotropic) non-vacuum solutions known [1], including both black hole and non-black hole solutions. Some recent special non-trivial non-vacuum exact solutions include those presented in [23] and [24], and especially solutions in teleparallel Born–Infeld inspired theories [25] (and [24]), solutions with electric and magnetic charges [23, 24] and solutions with scalar fields including the scalarized black hole solutions of [23, 26] (also see [22, 27]).

## Symmetries and *F*(*T*) teleparallel gravity

### Affine frame symmetries with isotropy

In Riemannian geometry in which the torsion and non-metricity are zero, the geometry is characterized by the metric $g_{ab}$ and its corresponding Levi–Civita connection, ${\overset{\circ }{\omega }}{}^a_{~bc}$. If $\textbf{X}$ is the infinitesimal generator of a given symmetry, then the symmetry must satisfy Killing’s equations (eqns.):

1a $$\begin{aligned} \mathcal {L}_{\textbf{X}} g_{ab}&= 0, \end{aligned}$$

1b $$\begin{aligned} \mathcal {L}_{\textbf{X}} {\overset{\circ }{\omega }}{}^a_{~bc}&= 0, \end{aligned}$$

where $\mathcal {L}$ is the Lie derivative. However Eq. (1b) is identically satisfied by definition of the Levi–Civita connection as it is computed from the metric. We also note that Eq. (1) are invariant under local Lorentz transformations $\Lambda ^a_{~b}$ of an orthonormal frame, the remaining gauge freedom present in Riemannian geometries.

The class of geometries in which the non-metricity and curvature tensors are zero is a *teleparallel geometry*. In such geometries, the geometry is characterized by the co-frame ${\textbf{h}}^a$ and a purely inertial Lorentz spin connection ${{{\varvec{\omega }}}}^a_{~b}$ [3, 8]. In Riemannian geometry, we see that the spin connection is computed from the metric, whereas in teleparallel geometry there exists some Lorentz transformation $\Lambda ^a_{~b}$ such that

2 $$\begin{aligned} \omega ^a_{~bc}=(\Lambda ^{-1})^a_{~d}{\textbf{h}}_c\left( \Lambda ^d_{~b}\right) . \end{aligned}$$

Since we have assumed an orthonormal frame, the geometry is invariant under Lorentz transformations, and therefore the spin connection ${{{\varvec{\omega }}}}^a_{~b}$ is not a dynamical quantity, and is only identically zero in a very special class of frames called *Proper Frames* [3, 8]. Clearly one cannot use Killings Eq. (1) to define or fix the symmetry. Any symmetry requirements needs to take into account the non-trivial nature of the spin connection resulting from the invariance of the geometry under Lorentz transformations.

Symmetries of a geometry where frames are the primary object describing the geometry have been previously explored in [28–30]. A complication arises when the group of symmetries contains a non-trivial linear isotropy group. If there is non-trivial linear isotropy group, then the determination of the group of symmetries necessarily requires the solution to a system of inhomogeneous differential equations (DEs) [31]:

3a $$\begin{aligned} \mathcal {L}_{\textbf{X}} {\textbf{h}}^{a}&= \lambda ^a_{~b} {\textbf{h}}^b, \end{aligned}$$

3b $$\begin{aligned} \mathcal {L}_{\textbf{X}} \omega ^a_{~bc}&= 0, \end{aligned}$$

with unknown local functions $\lambda ^a_{~b}=\lambda ^a_{~b}(x^\mu )$ which are elements of the Lie algebra of Lorentz transformations. We note that if the linear isotropy group is trivial [11], then $\lambda ^a_{~b} = 0$.

From Eqs. (3a) and (3b) it follows that the Lie derivative of the torsion tensor and its covariant derivatives with respect to $\textbf{X}$ are identically zero. We will call any vector field $\textbf{X}$ satisfying Eqs. (3a) and (3b) an *affine frame symmetry* of the geometry. We note that (3a) implies Eq. (1) while the second condition (3b) is the definition for an affine collineation [32].

A novel approach to explore the symmetries of any Riemann–Cartan geometry characterized by the frame and a metric compatible spin connection was proposed in [16]. In such Riemann–Cartan geometries, the connection is an independent object, and only if the curvature is zero does it reduce to teleparallel geometries. The approach in [16] relies on the existence and determination of a class of invariantly defined frames, which facilitates the solving the DEs (3) by fixing the functions $\lambda ^a_{~b}$ in an invariant way.

The Cartan–Karlhede (CK) algorithm [11, 33] provides a mechanism to determine a class of invariantly defined co-frames, ${\textbf{h}}^a$, up to the linear isotropy $H_q$ present in the geometry. An initial frame is chosen and the parameters of the Lorentz frame transformations $\lambda ^a_{~b}$ are fixed by normalizing the components of the torsion tensor and its covariant derivatives in an invariant manner. The CK algorithm provides a finite number of invariants that can be employed to determine the dimension of the symmetry group and locally characterize a geometry uniquely. One sequence of discrete invariants are of particular importance; the dimension of the linear isotropy group, $dim~H_{p}$. The linear isotropy group, $H_p$ is a subgroup of the Lorentz frame transformations that leave the torsion tensor and its covariant derivatives up to *p*th order invariant.

We summarize the approach in [16] that determines the class of invariantly define co-frames that can be employed as an ansatz for the characterization of the geometry satisfying an assumed group of symmetries. Consider a Riemann–Cartan geometry characterized by $({{\textbf{h}}}^a, {{{\varvec{\omega }}}}^a_{~b})$ that admits an affine frame symmetry generated by the vector field $\textbf{X}$. The class of *symmetry frames* are defined as the frames ${\textbf{h}}^a$ that satisfy

4 $$\begin{aligned} \mathcal {L}_{\textbf{X}} {\textbf{h}}^a = f_{X}^{~\hat{i}} \lambda ^{~a}_{\hat{i}~b} {\textbf{h}}^b, \end{aligned}$$

where $\lambda ^{~a}_{\hat{i}~b}$ are basis elements of the Lie algebra of the isotropy group ($\hat{i}$ ranges from 1 to the dimension of the isotropy group). The components of $f_X^{~\hat{i}}$ are local functions that depend only on the coordinates affected by the symmetry. This is a general definition and is not (strictly speaking) an invariantly defined frame until the components $f_{ X}^{~\hat{i}}$ are fixed in some coordinate independent way. The linear isotropy group associated with fixing the frame using Eq. (4) is given a new symbol, $\bar{H}_q$ in order to distinguish it from the linear isotropy group of the Cartan–Karlhede algorithm. This distinction is important as the elements of $\bar{H}_q$ are more restricted than their counterparts in $H_q$.

Since the components of $f_X^{~\hat{i}}$ are associated with the Lie derivative of a symmetry generating vector field, they are tensorial in nature that depend on the co-frame. Through the use of the CK algorithm we can judiciously choose the functions in $f_X^{~\hat{i}}$ to construct invariantly defined frames up to some minimal subgroup of the isotropy group. For example, if the geometry is spherically symmetric with the usual spherical coordinates $(t,r,\theta ,\phi )$, a choice for $f^{~\hat{i}}_{{X}_I}$, $I=1,2,3$, can be made so that the linear isotropy subgroup, $\bar{H}_q$, consists only of rotations in the subspace $\theta =\theta _0$ and $\phi =\phi _0$. In order to preserve the form of $f^{~\hat{i}}_{{X}_I}$ the Lorentz parameters are dependent on *t* and *r* only. As this procedure develops invariantly defined frames up to the linear isotropy group in Riemann–Cartan geometries, all that remains to restrict to teleparallel geometries is to solve the zero curvature constraint. In [16] we outlined a detailed procedure to fix the components of $f_X^{~\hat{i}}$ and determine $\overline{H}_q$ for the spherically symmetric geometries explicitly.

#### Determining the geometry for a group of affine symmetries with isotropy

The DEs that determine the most general teleparallel geometry which has a non-trivial linear isotropy group is presented in [16]. Here we quote Theorem III.2 in [16] for reference. The most general teleparallel geometry which admits a given group of affine frame symmetries, $\textbf{X}_I,~ I,J,K \in \{1, \ldots N\}$ with a non-trivial isotropy group of dimension *n* can be determined by solving for the unknowns $h^a_{~\mu }$, $f_I^{~\hat{i}}$ (with $\hat{i}, \hat{j}, \hat{k} \in \{1, \ldots n\}$) and $\omega ^a_{~bc}$ from the eqns.

5a $$\begin{aligned}&X_I^{~\nu } \partial _{\nu } h^a_{~\mu } + \partial _{\mu } X_I^{~\nu } h^a_{~\nu } = f_I^{~\hat{i}} \lambda ^a_{\hat{i}~b} h^b_{~\mu }, \end{aligned}$$

5b $$\begin{aligned}&2X_{[I} ( f_{J]}^{~\hat{k}}) - f_I^{~\hat{i}} f_J^{~\hat{j}} C^{\hat{k}}_{~\hat{i} \hat{j}} = C^K_{~IJ} f_K^{~\hat{k}}, \end{aligned}$$

5c $$\begin{aligned}&X_I^{~d} {\textbf{h}}_d( \omega ^a_{~bc}) + \omega ^d_{~bc} f_I^{~\hat{i}} \lambda ^a_{\hat{i}~d} - \omega ^a_{~dc} f_I^{~\hat{i}} \lambda ^d_{\hat{i}~b} \nonumber \\&\quad - \omega ^a_{~bd} f_I^{~\hat{i}} \lambda ^d_{\hat{i}~c} {\textbf{h}}_c( f_I^{~\hat{i}}) \lambda ^a_{\hat{i}~b} = 0, \end{aligned}$$

where $\{ \lambda ^a_{\hat{i}~b}\}_{\hat{i}=1}^n$ are a basis of the Lie algebra of the isotropy group, $[\lambda _{\hat{i}}, \lambda _{\hat{j}}] = C^{\hat{k}}_{~\hat{i}\hat{j}} \lambda _{\hat{k}}$, and $[\textbf{X}_I, \textbf{X}_J] = C^K_{~IJ} \textbf{X}_K$. Along with the additional condition that the curvature tensor expressed in terms of the spin connection and its derivatives must vanish in teleparallel geometries

6 $$\begin{aligned} R^a_{~bcd}=2\omega ^a_{~b[d,c]}+2\omega ^a_{~e[c}\omega ^e_{~bd]} +\omega ^a_{~be}c^e_{~cd} = 0. \end{aligned}$$

Note the equivalent of Eq. (6) in [16] has been corrected here to include the contribution of the anholonimity $c^e_{~cd}= 2h^e_{~[\mu ,\nu ]}h_c^{~\nu }h_d^{~\mu }$ to the expression for the curvature which is missing.

### Brief overview of *F*(*T*) teleparallel gravity

The complete Lagrangian for *F*(*T*) teleparallel gravity is

7 $$\begin{aligned} L= \frac{h}{2\kappa }F(T)+L_{Matt} \end{aligned}$$

where $\kappa$ is the gravitational coupling constant. The torsion scalar, *T*, can be expressed in terms of the torsion and the super-potential, $S^a_{~\mu \nu }$, as follows

8 $$\begin{aligned} T=\frac{1}{2}T^a_{~~\mu \nu }S_a^{~\mu \nu }, \end{aligned}$$

where

9 $$\begin{aligned} S_a^{~\mu \nu }=\frac{1}{2}\left( T_{a}^{~\mu \nu }+T^{\nu \mu }_{~~~~a} -T^{\mu \nu }_{~~~~a}\right) -h_a^{~\nu }T^{\phi \mu }_{~~~~\phi } + h_a^{\mu }T^{\phi \nu }_{~~~~\phi }. \nonumber \\ \end{aligned}$$

Assuming an orthonormal frame in which the tangent frame metric is $\eta _{ab}=Diag[-1,1,1,1]$, variations of the Lagrangian, which include a non-trivial spin connection [8, 34], yield *Lorentz covariant* FEs. Using Lagrange multipliers to impose the conditions that the spin connection is both metric compatible and flat, and performing the necessary variations with respect to these two multipliers, yields the following

10 $$\begin{aligned} \omega _{(ab)\mu } = 0 \ \hbox {and}\ \omega ^a_{~b\mu } = (\Lambda ^{-1})^a_{~c}\partial _\mu \Lambda ^c_{~b}. \end{aligned}$$

Since we have chosen an orthonormal gauge, there is a $\Lambda ^a_{b}\in SO(1,3)$ for which this is true.

The canonical energy momentum is defined as

11 $$\begin{aligned} h\varTheta _a^{~\mu }=-\frac{\delta L_{Matt}}{\delta h^{a}_{~\mu }}. \end{aligned}$$

The invariance of the FEs under Lorentz transformations implies that the canonical energy momentum is symmetric and it can be shown that the usual metrical energy momentum $T_{ab}$ is related to the symmetric part of the canonical energy momentum, that is,

12 $$\begin{aligned} \varTheta _{[ab]}=0,\quad \varTheta _{(ab)}= T_{ab}=-\frac{1}{2}\frac{\delta L_{Matt}}{\delta g_{ab}}. \end{aligned}$$

Variations of the Lagrangian, Eq. (7), with respect to the co-frame yield FEs that can be decomposed into symmetric and antisymmetric parts as

13a $$\begin{aligned} \kappa \varTheta _{(ab)}&= F''(T)S_{(ab)}^{~~~~\nu } \partial _{\nu } T+F'(T){\overset{\ \circ }{G}}{}_{ab} \nonumber \\&\quad + \frac{1}{2}g_{ab}\left( F(T)-TF'(T)\right) , \end{aligned}$$

13b $$\begin{aligned} 0&= F''(T)S_{[ab]}^{~~~~\nu } \partial _{\nu } T, \end{aligned}$$

where ${\overset{\ \circ }{G}}{}_{ab}$ is the Einstein tensor computed from the Levi–Civita connection of the metric.

From Eq. (13) we observe that if $T=T_0$, a constant, then the FEs for *F*(*T*) teleparallel gravity are equivalent to a rescaled version of TEGR (which looks like GR with a cosmological constant and a re-scaled coupling constant) [8]. In the case of TEGR, where $F(T)=T$, Eq. (13b) is identically satisfied, and again the theory reduces to a theory of gravity that is dynamically equivalent to GR. In general, if *F*(*T*) is non-linear, and *T* is not a constant, then the anti-symmetric part of the FEs (13b) impose constraints on the geometry [35–38].

## Spherical symmetry

Working in coordinates $x^\mu = (t, r, \theta , \phi )$, the affine frame symmetry generators associated with the three-dimensional spherical symmetry group are:

14 $$\begin{aligned} X_z= & {} \partial _{\phi },~X_y = - \cos \phi \partial _{\theta } + \frac{\sin \phi }{\tan \theta } \partial _{\phi }, X_x \nonumber \\= & {} \sin \phi \partial _{\theta } + \frac{\cos \phi }{\tan \theta } \partial _{\phi }. \end{aligned}$$

Writing $\{ X_I\}_{I=1}^3 = \{ X_x, X_y, X_z\}$ the non-zero commutator (structure) constants, $C^I_{~JK} = -\epsilon ^I_{~JK}$, are $C^{{3}}_{~{1} {2}} = -1,~ C^{{2}}_{~{1} {3}} = 1,~C^{{1}}_{~{2} {3}} = -1$. The basis for the isotropy group is of the form:

15 $$\begin{aligned}{} & {} \lambda _{\hat{1}} = \left[ \begin{array}{cccc} 0 &{} 0 &{} 0 &{} 0 \\ 0 &{} 0 &{} 0 &{} 0 \\ 0 &{} 0 &{} 0 &{} 1 \\ 0 &{} 0 &{} -1 &{} 0 \end{array} \right] ,~ \lambda _{\hat{2}} = -\left[ \begin{array}{cccc} 0 &{} 0 &{} 0 &{} 0 \\ 0 &{} 0 &{} 1 &{} 0 \\ 0 &{} -1 &{} 0 &{} 0 \\ 0 &{} 0 &{} 0 &{} 0 \end{array} \right] ,\nonumber \\{} & {} \lambda _{\hat{3}} = -\left[ \begin{array}{cccc} 0 &{} 0 &{} 0 &{} 0 \\ 0 &{} 0 &{} 0 &{} 1 \\ 0 &{} 0 &{} 0 &{} 0 \\ 0 &{} -1 &{} 0 &{} 0 \end{array} \right] , \end{aligned}$$

where the group $\overline{Iso}$ consists of all spatial rotations. Since $X_3 = X_z$ is a generator of a spatial rotation, we can choose our frame so that $X_3$ acts as a rotation on the basis elements ${\textbf{h}}^3$ and ${\textbf{h}}^4$ [16]; i.e.,

$$\begin{aligned} f_{3}^{~\hat{i}} \lambda _{\hat{i}} = \left[ \begin{array}{cccc} 0 &{} 0 &{} 0 &{} 0 \\ 0 &{} 0 &{} 0 &{} 0 \\ 0 &{} 0 &{} 0 &{} f_3^{~\hat{1}} \\ 0 &{} 0 &{} -f_3^{~\hat{1}} &{} 0 \end{array} \right] , \end{aligned}$$

then by applying a rotation about ${\textbf{h}}^3$ and ${\textbf{h}}^4$, the remaining component, $f_3^{~\hat{1}}$, can be set to zero. Choosing $f_3^{~\hat{i}}$ in this way, and using Eq. (5a), we find the solution:

16 $$\begin{aligned} f_2^{~\hat{i}} = f_2^{~\hat{i}}(\theta ) \cos \phi + g_2^{~\hat{i}}(\theta ) \sin \phi ; ~~ f_1^{~\hat{i}} = \partial _{\phi } f_2^{~\hat{i}}. \end{aligned}$$

If we use Lorentz transformations that are dependent on $\theta$ alone we can, without loss of generality, apply a rotation to set the coefficients of $\cos \phi$ in $f_2^{~\hat{i}}$ to zero. Equation (5a) with $\textbf{X}_2$ and $\textbf{X}_1$ then gives:

17 $$\begin{aligned} \partial _{\theta } g_2^{~\hat{i}} + \tan (\theta ) g_2^{~\hat{i}} = 0; ~~ g_2^{~\hat{i}} = \frac{C_2^{~\hat{i}}}{\sin (\theta )}. \end{aligned}$$

This fully determines $f_1^{~\hat{i}}$ and $f_2^{~\hat{i}}$, as the first is the $\phi$-derivative of the other. Applying a rotation with constant parameters, we can set $f_2^{~\hat{2}}= f_2^{~\hat{3}} = 0$, without loss of generality we will choose the first component to be non-zero and set the arbitrary constant to one, giving the following matrix representation of the coefficients:

18 $$\begin{aligned} f_I^{~\hat{i}} = \left[ \begin{array}{ccc} \frac{\cos (\phi )}{\sin (\theta )} &{} 0 &{} 0 \\ \frac{\sin (\phi )}{\sin (\theta )} &{} 0 &{} 0 \\ 0 &{} 0 &{} 0 \end{array} \right] . \end{aligned}$$

By making this choice, we have effectively fixed the frame up to $\overline{H}_q$ which consists of rotations in the ${\textbf{h}}^3-{\textbf{h}}^4$ plane with parameters dependent on coordinates *t* and *r*.

Relative to the representation for the isotropy group in Eq. (15), we can solve Eq. (6) to determine the most general frame admitting this symmetry group. We can choose a new coordinate system to “diagonalize” the frame. The resulting vierbein is:

19 $$\begin{aligned} h^a_{~\mu } = \left[ \begin{array}{cccc} A_1(t,r) &{} 0 &{} 0 &{} 0 \\ 0 &{} A_2(t,r) &{} 0 &{} 0 \\ 0 &{} 0 &{} A_3(t,r) &{} 0 \\ 0 &{} 0 &{} 0 &{} A_3(t,r) \sin (\theta ) \end{array}\right] . \end{aligned}$$

The form of the $f_I^{~\hat{i}}$ in Eq. (18) has been defined invariantly. With this choice the frame in Eq. (19) is an invariant symmetry frame, and we can now obtain the most general metric compatible connection:

20 $$\begin{aligned} \begin{aligned} \omega _{341}&= W_1(t,r),\qquad \qquad \qquad \omega _{342} = W_2(t,r), \\ \omega _{233}&= \omega _{244} = W_3(t,r), \\ \omega _{234}&= -\omega _{243} = W_4(t,r), \quad \omega _{121} = W_5(t,r),\\ \omega _{122}&= W_6(t,r),\\ \omega _{133}&= \omega _{144} = W_7(t,r),\quad \quad \omega _{134} = -\omega _{143} = W_8(t,r), \\ \omega _{344}&= - \frac{\cos (\theta )}{A_3 \sin (\theta )}. \end{aligned} \end{aligned}$$

Finally, to determine the most general connection for a teleparallel geometry we must impose the flatness condition in Eq. (6). The resulting eqns. can be solved so that any spherically symmetric teleparallel geometry is defined by (the three arbitrary functions in the vierbein (19) along with) the following spin connection components:

21 $$\begin{aligned} \begin{aligned} W_1&= -\frac{\partial _t \chi }{A_1} ,\qquad \qquad \quad \, W_2 = -\frac{\partial _r \chi }{A_2}, \\ W_3&= \frac{\cosh (\psi )\cos (\chi )}{A_3}, W_4 = \frac{\cosh (\psi )\sin (\chi )}{A_3},\\ W_5&= -\frac{\partial _t \psi }{A_1},\qquad \qquad \quad W_6 = -\frac{\partial _r \psi }{A_2},\\ W_7&= \frac{\sinh (\psi ) \cos (\chi )}{A_3}, W_8 = \frac{\sinh (\psi ) \sin (\chi )}{A_3}, \end{aligned} \end{aligned}$$

where $\chi$ and $\psi$ are arbitrary functions of the coordinates *t* and *r*.

Any choice of the arbitrary functions, $\psi$ and $\chi$, picks out a unique teleparallel geometry, as any change in the form of the spin connection which could affect the form of $\psi$ or $\chi$ leads to a change in the form of the vierbein. For a given pair of functions, the invariantly defined frame up to the linear isotropy group $\bar{H}_q$ arising from the CK algorithm could be computed to provide further sub-classification. We note that there are only five (the minimum number of) arbitrary functions required to specify a geometry: $A_1, A_2, A_3, \psi$ and $\chi$. We also note that there are four inequivalent branches of spherically symmetric teleparallel geometries, depending on whether $\chi$ or $\psi$ vanish when solving the antisymmetric FE, as the frame in Eq. (19) has been fully fixed by restricting the vierbein to a particular form. We note that special subclasses of these teleparallel geometries have been studied earlier in teleparallel gravity [39] using the Killing eqns. for an arbitrary spherically symmetric metric and using the non-invariant proper frame approach [10, 13, 40].

### Antisymmetric FEs

The antisymmetric FEs for the frame given by Eq. (19) and the connection given by Eq. (20) with $W_i$ given by Eq. (21) are:

22a $$\begin{aligned} 0= & {} \frac{F''\left( T\right) }{\kappa \,A_1\,A_2\,A_3}\,\left[ \partial _r\,T\,\left( A_1\,\cos \,\chi \,\sinh \,\psi +\partial _t\,A_3\right) \right. \nonumber \\{} & {} \left. -\partial _t\,T\,\left( A_2\,\cos \,\chi \,\cosh \,\psi +\partial _r\,A_3\right) \right] , \quad \end{aligned}$$

22b $$\begin{aligned} 0= & {} \frac{F''\left( T\right) \,\sin \,\chi }{\kappa \,A_1\,A_2\,A_3}\,\left[ A_1\,\cosh \,\psi \, \partial _r\,T-A_2\,\sinh \,\psi \,\partial _t\,T\right] , \nonumber \\ \end{aligned}$$

where *T* is the torsion scalar. There are two possible solutions:

- First case: $\sin \,\chi =0$: $\chi = n\,\pi$ where $n \in \,\mathbb {Z}$ is an integer and $\cos \,\chi =\cos \left( n\,\pi \right) =\pm 1 = \delta$, and
  23 $$\begin{aligned} \left( \partial _t\,T\right) = \left[ \frac{\delta \,A_1\,\sinh \,\psi +\partial _t\,A_3}{\delta \,A_2\,\cosh \,\psi +\partial _r\,A_3}\right] \left( \partial _r\,T\right) . \end{aligned}$$
- Second case: $$A_1\,\cosh \,\psi \,\partial _r\,T=A_2\,\sinh \,\psi \,\partial _t\,T$$.

There is a third case in which $T=\text {const}$, whence the antisymmetric FEs (22a) and (22b) are satisfied trivially since $\partial _t\,T=0$ and $\partial _r\,T=0$. Hence in this case the non-trivial symmetric FE reduce to those of GR, with a rescaled gravitational constant and a cosmological constant. We shall not be interested in such solutions here.

### Symmetric FEs for first case

There are four non-trivial components to the symmetric FEs: the three independent diagonal components (1,1), (2,2) and (3,3), and (1,2). On the diagonal, we obtained three independent and non-trivial eqns. because the components (3,3) and (4,4) are identical. However, we can also transform these four eqns. to obtain simpler eqns. This boils down to the eqns. ((2,2)–(3,3)), ((1,1)+(2,2)), (1,1) and (1,2) by substituting in Eq. (23). If we set $F\left( T\right) =T$, $F'\left( T\right) =1$ and $F''\left( T\right) =0$ for the TEGR case, the FEs will simplify further.

Before we attempt to solve the symmetric FEs for $T\left( t,\,r\right)$ and the $A_j\left( t,\,r\right)$ (an alternative form of the full FE is presented in Appendix A), let us express them in a simple way as follows:

24a $$\begin{aligned}{} & {} 0 = -F''\left( T\right) \,\left( \partial _r\,T\right) \,k_1\left( t,r\right) +F'\left( T\right) \,g_1\left( t,r\right) , \end{aligned}$$

24b $$\begin{aligned}{} & {} \kappa \,\left[ \rho +P\right] = -2\,F''\left( T\right) \,\left( \partial _r\,T\right) \,k_2\left( t,r\right) +2\,F'\left( T\right) \,g_2\left( t,r\right) , \nonumber \\ \end{aligned}$$

24c $$\begin{aligned}{} & {} \left[ \kappa \,\rho +\frac{F\left( T\right) }{2}\right] = -2\,F''\left( T\right) \,\left( \partial _r\,T\right) \,k_3\left( t,r\right) +2\,F'\left( T\right) \,g_3\left( t,r\right) , \nonumber \\ \end{aligned}$$

24d $$\begin{aligned}{} & {} 0 = -F''\left( T\right) \,\left( \partial _r\,T\right) \,k_4\left( t,r\right) +F'\left( T\right) \,g_4\left( t,r\right) , \end{aligned}$$

where the expressions $k_i$ and $g_i$ ($i=1\,-\,4$) are given explicitly by:

25a $$\begin{aligned} g_1&= \frac{1}{A_1^3\,A_2^3\,A_3^2}\Bigg [-A_1^2\,A_2\,A_3^2\,\left( \partial _r^2\,A_1\right) +A_1\,A_2^2\,A_3^2\, \left( \partial _t^2\,A_2\right) \nonumber \\&\quad -A_1^3\,A_2\,A_3\, \left( \partial _r^2\,A_3\right) -A_1\,A_2^3\,A_3\,\left( \partial _t^2\,A_3\right) \nonumber \\&\quad +A_1^3\,A_2\,\left( \partial _r\,A_3\right) ^2-A_1^3\,A_2^3\nonumber \\&\quad +\partial _r\,\left( A_1\,A_2\right) \,A_1^2\,A_3\, \left( \partial _r\,A_3\right) -A_1\,A_2^3\,\left( \partial _t\,A_3\right) ^2\nonumber \\&\quad +A_2^2\,A_3\,\partial _t\,\left( A_1\,A_2\right) \left( \partial _t\,A_3\right) \nonumber \\&\quad +A_1^2\,A_3^2 \left( \partial _r\,A_1\right) \left( \partial _r\,A_2\right) -A_2^2\,A_3^2\left( \partial _t\,A_1\right) \left( \partial _t\,A_2\right) \Bigg ] , \end{aligned}$$

25b $$\begin{aligned} g_2&= \frac{1}{A_1^3\,A_2^3\,A_3}\Bigg [-A_1^3\,A_2\,\left( \partial _r^2\,A_3\right) -A_1\,A_2^3\,\left( \partial _t^2\,A_3\right) \nonumber \\&\quad +\partial _r\,\left( A_1\,A_2\right) \,A_1^2\, \left( \partial _r\,A_3\right) +A_2^2\,\partial _t\,\left( A_1\,A_2\right) \left( \partial _t\,A_3\right) \Bigg ] , \end{aligned}$$

25c $$\begin{aligned} g_3&= \frac{1}{A_1^2\,A_2^3\,A_3^2}\Bigg [-A_1^2\,A_2\,A_3\,\left( \partial _r^2\,A_3\right) \nonumber \\&\quad -A_1^2\,A_2\,\left( \partial _r\,A_3\right) ^2-A_1\,A_2\,A_3\left( \partial _r\,A_3\right) \left( \partial _r\,A_1\right) \nonumber \\&\quad +A_1\,A_2^2\,A_3\left( \partial _r\,A_3\right) \left( \partial _t\,\psi \right) \nonumber \\&\quad -\delta \,A_1^2\,A_2^2\, \left( \partial _r\,A_3\right) \,\cosh \,\psi +A_1^2\,A_3\,\left( \partial _r\,A_2\right) \left( \partial _r\,A_3\right) \nonumber \\&\quad +A_2^3\,\left( \partial _t\,A_3\right) ^2+2A_2^2\,A_3\left( \partial _t\,A_2\right) \left( \partial _t\,A_3\right) \nonumber \\&\quad +\delta \,A_1\,A_2^3\left( \partial _t\,A_3\right) \, \sinh \psi -A_1\,A_2^2\,A_3\,\left( \partial _t\,A_3\right) \left( \partial _r\,\psi \right) \nonumber \\&\quad -A_1\,A_2^2\,A_3\left( -\delta \,A_2\cosh \psi \,\left( \partial _t\,\psi \right) +\delta \,A_1\,\sinh \psi \left( \partial _r\,\psi \right) \right. \nonumber \\&\quad \left. +\delta \,\cosh \psi \,\left( \partial _r\,A_1\right) -\delta \,\sinh \psi \,\left( \partial _t\,A_2\right) \right) \Bigg ] , \end{aligned}$$

25d $$\begin{aligned} g_4&= \frac{1}{A_1^2\,A_2^2\,A_3}\Bigg [-A_1\,A_2\left( \partial _r\,\partial _t\,A_3\right) +A_1\, \left( \partial _r\,A_3\right) \left( \partial _t\,A_2\right) \nonumber \\&\quad +A_2\,\left( \partial _r\,A_1\right) \left( \partial _t\,A_3\right) \Bigg ] , \end{aligned}$$

25e $$\begin{aligned} k_1&= \frac{1}{A_1^3\,A_2^3\,A_3^2}\Bigg [\left[ \frac{\delta \,A_1\,\sinh \,\psi +\partial _t\,A_3}{\delta \,A_2\,\cosh \,\psi +\partial _r\,A_3}\right] \Bigg [A_1\,A_2^3\,A_3\nonumber \\&\quad \times \left( \partial _t\,A_3\right) +A_1\,A_2^2\,A_3\,\Bigg (-A_3\,\left( \partial _t\,A_2\right) +A_1\nonumber \\&\quad \times \Bigg (\delta \,A_2\,\sinh \psi +A_3\, \left( \partial _r\,\psi \right) \Bigg )\Bigg )\Bigg ]\nonumber \\&\quad +\Bigg [A_1^3\,A_2\,A_3\,\left( \partial _r\,A_3\right) -A_1^2\,A_2\,A_3\left( -A_3\,\left( \partial _r\,A_1\right) \right. \nonumber \\&\quad \left. +A_2\,\left( -\delta \,A_1\,\cosh \psi +A_3\,\left( \partial _t\,\psi \right) \right) \right) \Bigg ]\Bigg ] , \end{aligned}$$

25f $$\begin{aligned} k_2&= \frac{1}{A_1^2\,A_2^2\,A_3}\nonumber \\&\quad \times \left[ \frac{A_2^2\,\left[ \delta \,A_1\,\sinh \,\psi +\partial _t\,A_3\right] ^2 +A_1^2\left[ \partial _r\,A_3+\delta \,A_2\,\cosh \psi \right] ^2}{\delta \,A_2\,\cosh \,\psi +\partial _r\,A_3}\right] , \end{aligned}$$

25g $$\begin{aligned} k_3&= \frac{1}{A_2^2\,A_3}\left[ \delta \,A_2\,\cosh \psi +\partial _r\,A_3\right] , \end{aligned}$$

25h $$\begin{aligned} k_4&= \frac{1}{A_1\,A_2\,A_3}\left[ \delta \,A_1\,\sinh \,\psi +\partial _t\,A_3\right] . \end{aligned}$$

Equations (24a) and (24d) yield the following relation without $F\left( T\right)$ and its derivatives:

26 $$\begin{aligned} g_1\left( t,r\right) \,k_4\left( t,r\right) = g_4\left( t,r\right) \,k_1\left( t,r\right) , \end{aligned}$$

which can be written explicitly as:

27 $$\begin{aligned}{} & {} \Bigg [-A_1^2\,A_2\,A_3^2\,\left( \partial _r^2\,A_1\right) +A_1\,A_2^2\,A_3^2\, \left( \partial _t^2\,A_2\right) -A_1^3\,A_2\,A_3\nonumber \\{} & {} \quad \quad \times \left( \partial _r^2\,A_3\right) -A_1\,A_2^3\,A_3\, \left( \partial _t^2\,A_3\right) +A_1^3\,A_2\,\left( \partial _r\,A_3\right) ^2\nonumber \\{} & {} \quad \quad -A_1^3\,A_2^3+\partial _r\,\left( A_1\,A_2\right) \,A_1^2\,A_3\,\left( \partial _r\,A_3\right) \nonumber \\{} & {} \quad \quad -A_1\,A_2^3\,\left( \partial _t\,A_3\right) ^2+A_2^2\,A_3\,\partial _t\,\left( A_1\,A_2\right) \left( \partial _t\,A_3\right) \nonumber \\{} & {} \quad \quad +A_1^2\,A_3^2\left( \partial _r\,A_1\right) \left( \partial _r\,A_2\right) -A_2^2\,A_3^2 \left( \partial _t\,A_1\right) \left( \partial _t\,A_2\right) \Bigg ]\nonumber \\{} & {} \quad \quad \times \Bigg (A_1\,A_2\,\left[ \delta \,A_1\,\sinh \,\psi +\partial _t\,A_3\right] \Bigg )\nonumber \\{} & {} \quad =\Bigg [\left[ \frac{\delta \,A_1\,\sinh \,\psi +\partial _t\,A_3}{\delta \,A_2\,\cosh \, \psi +\partial _r\,A_3}\right] \nonumber \\{} & {} \quad \quad \times \Bigg [A_1\,A_2^3\,A_3\,\left( \partial _t\,A_3\right) +A_1\,A_2^2\,A_3\,\Bigg (-A_3\,\left( \partial _t\,A_2\right) \nonumber \\{} & {} \quad \quad +A_1\,\Bigg (\delta \,A_2\,\sinh \psi +A_3\,\left( \partial _r\,\psi \right) \Bigg )\Bigg )\Bigg ]\nonumber \\{} & {} \quad \quad +\Bigg [A_1^3\,A_2\,A_3\,\left( \partial _r\,A_3\right) -A_1^2\,A_2\,A_3\,\left( -A_3\, \left( \partial _r\,A_1\right) \right. \nonumber \\{} & {} \left. \quad \quad +A_2\,\left( -\delta \,A_1\,\cosh \psi +A_3\, \left( \partial _t\,\psi \right) \right) \right) \Bigg ]\Bigg ]\nonumber \\{} & {} \quad \quad \times \Bigg (-A_1\,A_2\left( \partial _r\,\partial _t\,A_3\right) +A_1\,\left( \partial _r\,A_3\right) \left( \partial _t\,A_2\right) \nonumber \\{} & {} \quad \quad +A_2\,\left( \partial _r\,A_1\right) \left( \partial _t\,A_3\right) \Bigg ). \end{aligned}$$

This is a partial differential equation (PDE) involving only the $A_j\left( t,r\right)$ ($j = 1-3$) and $\psi \left( t,r\right)$.

Equations (24a) and (24d) also yield

28 $$\begin{aligned} \partial _r\,\left[ \ln \,F'\left( T\right) \right] =\frac{g_1\left( t,r\right) }{k_1\left( t,r\right) }, \end{aligned}$$

which can be formally integrated to find

29 $$\begin{aligned} F'\left( T\right) =C\left( t\right) \,\exp \left[ \int \,dr'\,\frac{g_1\left( t,r'\right) }{k_1\left( t,r'\right) }\right] , \end{aligned}$$

where $C\left( t\right)$ is an arbitrary function of *t* only. In principle, we can eventually solve this Eq. (29) for $F\left( T(t,r)\right)$. By substituting the Eqs. (28) and (29) in the Eqs. (24b) and (24c), we obtain the following:

30a $$\begin{aligned} \kappa \,\left[ \rho +P\right]= & {} 2\,C\left( t\right) \,\exp \left[ \int \,dr'\,\frac{g_1\left( t,r'\right) }{k_1\left( t,r'\right) }\right] \nonumber \\{} & {} \times \left[ g_2\left( t,r\right) -k_2\left( t,r\right) \,\left( \frac{g_1\left( t,r\right) }{k_1\left( t,r\right) }\right) \right] , \nonumber \\ \end{aligned}$$

30b $$\begin{aligned} \left[ \kappa \,\rho +\frac{F\left( T\right) }{2}\right]= & {} 2\,C\left( t\right) \, \exp \left[ \int \,dr'\,\frac{g_1\left( t,r'\right) }{k_1\left( t,r'\right) }\right] \nonumber \\{} & {} \times \left[ g_3\left( t,r\right) -k_3\left( t,r\right) \,\left( \frac{g_1\left( t,r\right) }{k_1\left( t,r\right) }\right) \right] . \nonumber \\ \end{aligned}$$

We may also replace $\frac{g_1\left( t,r'\right) }{k_1\left( t,r'\right) }$ terms by $\frac{g_4\left( t,r'\right) }{k_4\left( t,r'\right) }$ term inside Eqs. (30a) and (30b) for more simplicity.

The form of the torsion scalar becomes (using $\sin \,\chi =0$ and $\cos \,\chi = \delta$):

31 $$\begin{aligned} T=&\left( -4\,\delta \,{\frac{\sinh \left( \psi ( t,r)\right) }{A_2(t,r) A_3(t,r)}} -4\,{\frac{\partial _t\,A_3(t,r)}{A_2(t,r) A_3(t,r) A_{1} \left( t,r \right) }} \right) \nonumber \\&\times \partial _r\,\psi ( t,r) +4\,\delta \,{\frac{ \left( \partial _t\psi ( t,r)\right) \cosh \left( \psi ( t,r)\right) }{A_1(t,r) A_3(t,r)}}\nonumber \\&-4\,\delta \,{\frac{\left( {\partial _r}A_{1} \left( t,r \right) \right) \cosh \left( \psi ( t,r)\right) }{A_2(t,r) A_3(t,r) A_{1} \left( t,r \right) }}\nonumber \\&+4\,\delta \,{\frac{ \left( {\partial _t}A_2(t,r)\right) \sinh \left( \psi ( t,r)\right) }{A_2(t,r) A_3(t,r) A_1(t,r)}}\nonumber \\&-4\,\delta \,\frac{\cosh \left( \psi ( t,r)\right) {\partial _r}A_3(t,r)}{A_2(t,r)\left( A_3(t,r)\right) ^{2}}\nonumber \\&+4\,\delta \,{\frac{\sinh \left( \psi ( t,r) \right) {\partial _t}A_3(t,r) }{A_1(t,r) \left( A_3(t,r)\right) ^{2}}} +4\,{\frac{\left( {\partial _t}\psi ( t,r)\right) {\partial _r}A_3(t,r) }{A_2(t,r) A_3(t,r) A_{1} \left( t,r\right) }}\nonumber \\&-4\,{\frac{\left( {\partial _r}A_1(t,r)\right) {\partial _r}A_3(t,r) }{A_3(t,r) \left( A_2(t,r)\right) ^{2}A_1(t,r) }}\nonumber \\&+4\,{\frac{\left( {\partial _t}A_2(t,r)\right) {\partial _t}A_{3} \left( t,r \right) }{A_3(t,r)\left( A_1(t,r)\right) ^{2}A_2(t,r)}} -2\,{\frac{\left( {\partial _r}A_{3}\left( t,r \right) \right) ^{2}}{\left( A_2(t,r)\right) ^{2} \left( A_3(t,r) \right) ^{2}}}\nonumber \\&+2\,{\frac{\left( {\partial _t}A_{3}\left( t,r \right) \right) ^{2}}{\left( A_1(t,r)\right) ^{2} \left( A_3(t,r) \right) ^{2}}}-2\,\left( A_3(t,r)\right) ^{-2} . \end{aligned}$$

### Symmetric FEs for second case

For the general solution in this case, we substitute the Eq. (22b) into the Eq. (22a) for the antisymmetric FEs. We recall for $\partial _i\,T \ne 0$ that $\partial _t\,T=\frac{A_1\,\cosh \,\psi }{A_2\,\sinh \,\psi }\,\partial _r\,T$, so that:

32 $$\begin{aligned} \sinh \,\psi \,\left( A_1^{-1} \partial _t\,A_3\right) = \cos \,\chi +\cosh \,\psi \,\left( A_2^{-1} \partial _r\,A_3\right) . \nonumber \\ \end{aligned}$$

We can isolate $\chi \left( t,\,r\right)$ in the following way:

33 $$\begin{aligned} \cos (\chi \left( t,\,r\right) )= & {} \sinh \,\psi \,\left( A_1^{-1}\,\partial _t\,A_3\right) \nonumber \\{} & {} -\cosh \,\psi \,\left( A_2^{-1}\,\partial _r\,A_3\right) \end{aligned}$$

We have in Eq. (33) a relation giving $\chi$ as a function of $\psi$ and the other $A_j$ as a function of *t* and *r*. For the eqn. giving *T*, this will reduce to the following PDE:

34 $$\begin{aligned} \partial _t\,T = \left[ \frac{A_1\,\coth \,\psi }{A_2}\right] \,\left( \partial _r\,T\right) . \end{aligned}$$

There are also four non-trivial components to the symmetric FEs: three independent diagonals plus the component (1,2) because components (3,3) and (4,4) are identical. However, we have also transformed these four eqns. to obtain much simpler eqns. Before solving the FEs for $T\left( t,\,r\right)$ and the $A_j\left( t,\,r\right)$ in general, we express the symmetric FEs in the same manner as in Eqs. (24a)–(24d). The corresponding expressions for the functions $k_i$ and $g_i$ ($i=1\,-\,4$) are given in this case by:

35a $$\begin{aligned} g_1&= \frac{1}{A_1^3\,A_2^3\,A_3^2}\Bigg [-A_1^2\,A_2\,A_3^2\,\left( \partial _r^2\,A_1\right) +A_1\,A_2^2\,A_3^2\,\left( \partial _t^2\,A_2\right) \nonumber \\&\quad -A_1^3\,A_2\,A_3\,\left( \partial _r^2\,A_3\right) -A_1\,A_2^3\,A_3\,\left( \partial _t^2\,A_3\right) \nonumber \\&\quad +A_1^3\,A_2\,\left( \partial _r\,A_3\right) ^2+\partial _r\,\left( A_1\,A_2\right) \,A_1^2\,A_3\, \left( \partial _r\,A_3\right) \nonumber \\&\quad -A_1\,A_2^3\,\left( \partial _t\,A_3\right) ^2+A_2^2\,A_3\,\partial _t\, \left( A_1\,A_2\right) \left( \partial _t\,A_3\right) +A_1^2\,A_3^2\nonumber \\&\quad \times \left( \partial _r\,A_1\right) \left( \partial _r\,A_2\right) -A_2^2\,A_3^2\left( \partial _t\,A_1\right) \left( \partial _t\,A_2\right) -A_1^3\,A_2^3\Bigg ] , \end{aligned}$$

35b $$\begin{aligned} g_2&= \frac{1}{A_1^3\,A_2^3\,A_3} \Bigg [-A_1^3\,A_2\,\left( \partial _r^2\,A_3\right) -A_1\,A_2^3\, \left( \partial _t^2\,A_3\right) +\partial _r\nonumber \\&\quad \times \left( A_1\,A_2\right) \,A_1^2\,\left( \partial _r\,A_3\right) +A_2^2\,\partial _t\,\left( A_1\,A_2\right) \left( \partial _t\,A_3\right) \Bigg ] , \end{aligned}$$

35c $$\begin{aligned} g_3&= \frac{1}{A_1^3\,A_2^3\,A_3^2}\Bigg [A_1^3\,A_2\,A_3\,\left( \partial _r^2\,A_3\right) \, \left( \delta \,\cosh ^2\psi -1\right) \nonumber \\&\quad +A_1\,A_2^3\,A_3\,\delta \sinh ^2\psi \left( \partial _t^2\,A_3\right) \nonumber \\&\quad -2A_1^2\,A_2^2\,A_3\sinh \psi \,\cosh \psi \,\delta \,\left( \partial _r\,\partial _t\,A_3\right) \nonumber \\&\quad +A_1^3\,A_2\, \sinh ^2\psi \left( \partial _r\,A_3\right) ^2\nonumber \\&\quad -2A_1^2\,A_2^2\,\left( \partial _t\,A_3\right) \left( \partial _r\,A_3\right) \,\cosh \psi \,\sinh \psi \nonumber \\&\quad +A_1^2\,A_3\, \left( \partial _r\,A_3\right) \Bigg (-A_2^2\sinh ^2\psi \left( 1+\delta \right) \left( \partial _t\psi \right) +A_2\nonumber \\&\quad \times \left( \partial _r\,A_1\right) \sinh ^2\psi +A_2\,\left( \partial _t\,A_2\right) \left( \delta -1\right) \sinh \psi \,\cosh \psi \nonumber \\&\quad +A_1\,A_2\left( 1+\delta \right) \cosh \psi \,\sinh \psi \,\left( \partial _r\,\psi \right) -A_1\,\left( \partial _r\,A_2\right) \nonumber \\&\quad \times \left( \delta \cosh ^2\psi -1\right) \Bigg )+A_1\,A_2^3 \cosh ^2\psi \left( \partial _t\,A_3\right) ^2\nonumber \\&\quad -A_2^2\,A_3\left( \partial _t\,A_3\right) \Bigg (-A_1\,A_2\cosh \psi \,\sinh \psi \left( \delta +1\right) \left( \partial _t\psi \right) \nonumber \\&\quad -A_1\left( \partial _r\,A_1\right) \cosh \psi \,\sinh \psi \left( \delta -1\right) \nonumber \\&\quad -A_1\left( \partial _t\,A_2\right) \left( \cosh ^2\psi +1\right) +A_1^2\,\left( \delta +1\right) \nonumber \\&\quad \times \cosh ^2\psi \left( \partial _r\,\psi \right) +A_2\,\left( \partial _t\,A_1\right) \,\delta \,\sinh ^2\psi \Bigg )\Bigg ] , \end{aligned}$$

35d $$\begin{aligned} g_4&= \frac{1}{A_1^2\,A_2^2\,A_3}\Bigg [-A_1\,A_2\left( \partial _r\,\partial _t\,A_3\right) +A_1\,\left( \partial _r\,A_3\right) \left( \partial _t\,A_2\right) \nonumber \\&\quad +A_2\,\left( \partial _r\,A_1\right) \left( \partial _t\,A_3\right) \Bigg ] , \end{aligned}$$

35e $$\begin{aligned} k_1&= \frac{1}{A_1\,A_2^2\,A_3}\Bigg [\coth \,\psi \Bigg [\cosh \psi \left[ A_1\left( \partial _r\,A_3\right) \, \sinh \psi \right. \nonumber \\&\quad \left. -A_2\,\left( \partial _t\,A_3\right) \cosh \psi \right] -A_3\left( A_1\,\left( \partial _r\psi \right) -\left( \partial _t\,A_2\right) \right) \Bigg ]\nonumber \\&\quad +\Bigg [\sinh \psi \,\left[ A_1\,\left( \partial _r\,A_3\right) \,\sinh \psi -A_2\,\left( \partial _t\,A_3\right) \cosh \psi \right] \nonumber \\&\quad +A_3\,\left[ A_2\,\left( \partial _t\psi \right) -\left( \partial _r\,A_1\right) \right] \Bigg ]\Bigg ] , \end{aligned}$$

35f $$\begin{aligned} k_2&= -\frac{1}{A_1\,A_2^2\,A_3}\left( \frac{1+2\,\sinh ^2\psi }{\sinh \psi }\right) \left[ A_1\, \sinh \psi \left( \partial _r\,A_3\right) \right. \end{aligned}$$

35g $$\begin{aligned}&\quad \left. -A_2\,\cosh \psi \,\left( \partial _t\,A_3\right) \right] , \end{aligned}$$

35h $$\begin{aligned} k_3&= -\frac{\sinh \psi }{A_1\,A_2^2\,A_3} \left[ A_1\,\left( \partial _r\,A_3\right) \sinh \psi \right. \nonumber \\ {}&\quad \times \left. -A_2\,\cosh \psi \, \left( \partial _t\,A_3\right) \right] , \end{aligned}$$

35i $$\begin{aligned} k_4&= -\frac{1}{A_1\,A_2^2\,A_3}\,\cosh \psi \,[A_1\,\left( \partial _r\,A_3\right) \sinh \psi \nonumber \\&\quad -A_2\, \left( \partial _t\,A_3\right) \,\cosh \psi ] . \end{aligned}$$

We will obtain the same types of relations as Eqs. (30a) and (30b) for the above functions $k_i\left( t,r\right)$, and $g_i\left( t,r\right)$. The Eq. (26) represents a relation involving only the $A_j\left( t,r\right)$ and $\psi \left( t,r\right)$.

The torsion scalar is expressed in Eq (36).

36 $$\begin{aligned} T&= -4\,{\frac{\sinh \left( \psi ( t,r) \right) \left( {\partial _t}\arccos \left[ \sinh \,\psi \,\left( A_1^{-1}\, \partial _t\,A_3\right) -\cosh \,\psi \,\left( A_2^{-1}\,\partial _r\,A_3\right) \right] \right) }{A_{1} \left( t,r \right) A_3(t,r) }}\nonumber \\&\quad \times \sin \left( \arccos \left[ \sinh \,\psi \,\left( A_1^{-1}\,\partial _t\,A_3\right) -\cosh \,\psi \,\left( A_2^{-1}\,\partial _r\,A_3\right) \right] \right) \nonumber \\&\quad +4\,{\frac{\cosh \left( \psi ( t,r) \right) \left( {\partial _r}\arccos \left[ \sinh \,\psi \,\left( A_1^{-1}\, \partial _t\,A_3\right) -\cosh \,\psi \,\left( A_2^{-1}\,\partial _r\,A_3\right) \right] \right) }{A_2(t,r) A_3(t,r) }}\nonumber \\&\quad \times \sin \left( \arccos \left[ \sinh \,\psi \,\left( A_1^{-1}\,\partial _t\,A_3\right) -\cosh \,\psi \,\left( A_2^{-1}\,\partial _r\,A_3\right) \right] \right) \nonumber \\&\quad + \left( -4\,{\frac{\sinh \left( \psi ( t,r) \right) \left[ \sinh \,\psi \,\left( A_1^{-1}\,\partial _t\,A_3\right) -\cosh \,\psi \,\left( A_2^{-1}\,\partial _r\,A_3\right) \right] }{A_2(t,r) A_3(t,r) }} -4\,{\frac{{\partial _t}A_3(t,r) }{A_2(t,r) A_3(t,r) A_{1} \left( t,r \right) }} \right) \nonumber \\&\quad \times {\partial _r}\psi ( t,r)+4\,{\frac{ \left( {\partial _t}\psi ( t,r) \right) \cosh \left( \psi ( t,r) \right) \left[ \sinh \,\psi \,\left( A_1^{-1}\,\partial _t\,A_3\right) -\cosh \,\psi \,\left( A_2^{-1}\,\partial _r\,A_3\right) \right] }{A_1(t,r) A_3(t,r)}}\nonumber \\&\quad -4\,{\frac{ \left( {\partial _r}A_{1} \left( t,r \right) \right) \left[ \sinh \,\psi \, \left( A_1^{-1}\,\partial _t\,A_3\right) -\cosh \,\psi \,\left( A_2^{-1}\,\partial _r\,A_3\right) \right] \cosh \left( \psi ( t,r)\right) }{A_2(t,r) A_3(t,r) A_{1} \left( t,r \right) }}\nonumber \\&\quad +4\,{\frac{ \left( {\partial _t}A_2(t,r) \right) \left[ \sinh \,\psi \,\left( A_1^{-1}\, \partial _t\,A_3\right) -\cosh \,\psi \,\left( A_2^{-1}\,\partial _r\,A_3\right) \right] \sinh \left( \psi ( t,r) \right) }{A_2(t,r) A_3(t,r) A_1(t,r) }}\nonumber \\&\quad -4\,{\frac{\cosh \left( \psi ( t,r) \right) \left[ \sinh \,\psi \,\left( A_1^{-1}\,\partial _t\,A_3\right) -\cosh \,\psi \,\left( A_2^{-1}\,\partial _r\,A_3\right) \right] {\partial _r}A_3(t,r) }{A_2(t,r) \left( A_3(t,r) \right) ^{2}}}\nonumber \\&\quad +4\,{\frac{\sinh \left( \psi ( t,r) \right) \left[ \sinh \,\psi \,\left( A_1^{-1}\,\partial _t\,A_3\right) -\cosh \,\psi \,\left( A_2^{-1}\,\partial _r\,A_3\right) \right] {\partial _t}A_3(t,r) }{A_1(t,r) \left( A_3(t,r) \right) ^{2}}}\nonumber \\&\quad +4\,{\frac{ \left( {\partial _t}\psi ( t,r) \right) {\partial _r}A_3(t,r) }{A_2(t,r) A_3(t,r) A_{1} \left( t,r \right) }}-4\,{\frac{ \left( {\partial _r}A_1(t,r) \right) {\partial _r}A_3(t,r) }{A_3(t,r) \left( A_2(t,r) \right) ^{2}A_1(t,r) }}+4\,{\frac{ \left( {\partial _t}A_2(t,r) \right) {\partial _t}A_{3} \left( t,r \right) }{A_3(t,r) \left( A_1(t,r) \right) ^{2}A_2(t,r) }}\nonumber \\&\quad -2\,{\frac{ \left( {\partial _r}A_{3} \left( t,r \right) \right) ^{2}}{ \left( A_2(t,r) \right) ^{2} \left( A_3(t,r) \right) ^{2}}} +2\,{\frac{ \left( {\partial _t}A_{3} \left( t,r \right) \right) ^{2}}{ \left( A_1(t,r) \right) ^{2} \left( A_3(t,r) \right) ^{2}}}-2\, \left( A_3(t,r)\right) ^{-2}. \end{aligned}$$

Having found the general form of the spin connection, we can now in principle apply a Lorentz transformation to set the spin connection to zero and explicitly present the general spherically symmetric geometry in the proper frame.

#### Coordinate choice

There is a class of transformations that maintain the “diagonal” form of the frame in (19). But, in general, this will also lead to a change in the form of the connection and the FEs. Therefore, and in order to maintain the comoving nature of the perfect fluid source, we restrict ourselve to transformations of the form $t \rightarrow f(t)$ and $r \rightarrow g(r)$ (i.e., redefining the *r* and *t* coordinates). This may simplify the eqns. further. However, even then the explicit forms for, e.g., the symmetry vectors (and the similarity variable, *z* – see later) will change.

We shall look for solutions for particular functions *F*(*T*), with an eqn. of state $P=\alpha \,\rho$, and with additional symmetries. First, let us review some special geometries.

### Robertson–Walker geometries

In [16] we discussed the teleparallel Robertson–Walker (TRW) geometries with a 6D group of affine symmetries. Working in the coordinate system $(t, r, \theta , \phi )$ (and in a diagonal co-frame with $A_1=1,\,A_2=a(t)\,/{\sqrt{1-kr^2}},\, A_3=r\,a(t)$), the additional three affine frame symmetry generators (in addition to the three associated with spherical symmetry) of the RW metric were displayed and the most general metric-compatible connection was obtained. In the flat ($k=0$) case the non-trivial components are:

37 $$\begin{aligned} \omega _{233} = \omega _{244} = - \frac{1}{a(t)r}, \quad \quad \omega _{344} = - \frac{\cos (\theta )}{a(t) r \sin (\theta )}. \end{aligned}$$

The TRW class was determined using the proper frame approach in [41], which is not an invariant approach [41]. Many of the applications in the literature of these geometries have concentrated on the analysis of the flat ($k = 0$) TRW cosmological models [1, 2]. But TRW models with non-zero *k* have been studied [42, 43]. In addition, particular forms for *F*(*T*) have been considered [44].

### de Sitter geometry

We recall that in GR, the de Sitter solution is a special case of the flat FLRW solutions. Hence we consider the special subclass of flat $k = 0$ TRW geometries which admit a seven-dimensional symmetry group. This is given by the TRW vierbein and the connection where (the functions $\chi = \psi =0$ and) *a*(*t*) is defined by $a(t) = C_3 e^{C_4 t}$. In this case the (seventh) affine symmetry is given by

38 $$\begin{aligned} X = \frac{1}{C_0} \partial _t + r \partial _r. \end{aligned}$$

where $C_0 \equiv -H_0$. The teleparallel de Sitter geometry (TdS) is defined to be the teleparallel geometry with the $G_7$ Lie group of affine symmetries, $\mathcal {R} \rtimes E(3)$ which is a subgroup of *O*(1, 4). We note in this geometry the covariant derivative of the torsion tensor is zero.

### Equivalence of FLRW subclasses

Let us next show that the $k = +1$ and $k = -1$ cases are inequivalent and that the subcases with $\delta = \pm 1$ are equivalent using Cartan invariants [11]. For both signs of *k* the Cartan invariants appear as the components of the irreducible parts of the torsion tensor and their first covariant derivative. This is due to the fact that the linear isotropy group is *SO*(3) at all iterations of the CK algorithm, and that the number of functionally independent invariants is at most one.

1. $k = +1$: At zeroth order, the invariants are
  39 $$\begin{aligned} V_1 = - \frac{3 \dot{a}}{a},\quad A_1 = - \frac{2 \delta \sqrt{k}}{a}, \end{aligned}$$
  while at first order, we find the following two invariants
  40 $$\begin{aligned} (\nabla \textbf{V})_{11} = \dot{V_1}, \quad (\nabla \textbf{A})_{11} = - \frac{2 \delta \sqrt{k} \dot{a} }{a^2}. \end{aligned}$$
2. $k = -1$: At zeroth order, the sole invariant is
  41 $$\begin{aligned} V_1 = 3 \left( \frac{\dot{a}}{a} + \frac{\delta \sqrt{-k}}{a}\right) , \end{aligned}$$
  while at first order we find two invariants
  42 $$\begin{aligned} (\nabla \textbf{V})_{11}= & {} \dot{V_1},\nonumber \\ (\nabla \textbf{V})_{22}= & {} (\nabla \textbf{V})_{33} = (\nabla \textbf{V})_{44} = \frac{\delta \sqrt{-k} V_1}{a}. \end{aligned}$$

It is clear that the cases $k = \pm 1$ must be inequivalent due to the differing invariant structure of the Cartan invariants. It is less obvious, if the $\delta = \pm 1$ are equivalent.

Alternatively, the connection coefficients for this case can be determined using the connection coefficients of the spherically symmetric case in [45] where the vierbein is given by Eq. (19), while the spin-connection is given by Eq. (20) with the ansatz described by Eqs. (21). In the case of TRW symmetry, the affine frame symmetry conditions lead to further conditions on the $W_i$’s:

43a $$\begin{aligned} k<0{} & {} : \chi = 0, \pi ,~\psi = \cosh ^{-1} (-\cos (\chi ) \sqrt{1-kr^2}), \nonumber \\ \end{aligned}$$

43b $$\begin{aligned} k \ge 0{} & {} : \chi = \cos ^{-1} (- \sqrt{1-kr^2}), \psi = 0. \end{aligned}$$

This choice of representation of the arbitrary functions in the connection coefficients shows that the sign difference (i.e., $\delta =\pm 1$) in the case of $k >0$ is purely a choice of representation and hence these subcases are equivalent.

We note that in the $k<0$ case, $\delta = \cos (\chi )$ and the two subcases $\delta = \pm 1$ are not related by a proper orthochronous Lorentz transformation, since transformations of the form $r\,\rightarrow \,-r$ and $t \,\rightarrow \,-t$ are required to transform from one to the other. This implies that the two subcases are not equivalent under $SO^*\left( 1,\,3\right)$.

## Static spherical symmetric spacetimes

Within teleparallel gravity, stationary or static spherically symmetric geometries have been discussed in applications [39, 40, 46]. In these papers, the stationarity or static condition is not rigorously derived but instead arises from restricting the arbitrary functions to be dependent on the radial coordinate alone. Although such a choice intuitively makes sense, it does not necessarily constitute the most general class of stationary or static spherically symmetric geometries since it does not consider the inequivalent subclasses of spherically symmetric geometries.

Stationary spherically symmetric geometries can be produced by including an additional affine frame symmetry, $\textbf{Y}$ with $Y^a Y_a < 0$ locally. This vector field must commute with the original frame symmetries, $[\textbf{X}_I, \textbf{Y}] = 0$ for $I \in \{1, \ldots , 3\}$. Therefore, a new coordinate system can be chosen so that $\textbf{Y} = \partial _{t}$, while preserving the vierbein in *non-diagonal* form instead of the more specialized vierbein in Eq. (19); that is, in general we cannot use a coordinate transformation for stationary solutions because our vierbein ansatz is set to a diagonal metric and in the coordinate system where $\textbf{X} = \partial _t$ the metric will, in general, have a non-diagonal (i.e., *dtdr*) cross-term. The timelike condition on $\textbf{Y}$ imposes an additional condition on the vierbein functions $A_i$, $i\in \{1, \ldots , 3\}$.

Static spherically symmetric geometries are produced by requiring that $\textbf{Y}$ is hypersurface orthogonal:

44 $$\begin{aligned} \textbf{Y} \wedge d \textbf{Y} = 0. \end{aligned}$$

In the case of static solutions, the diagonal vierbein form (19) is applicable (unlike in the stationary case). The expansion of the symmetry group introduces three additional functions and this can be determined using (5a). As the commutator constants are zero, the analysis of these eqns. is straightforward and imply that the vierbein and the components of the connection one-form must be dependent on the coordinate *r* alone. As no other conditions are imposed, this proves that the static spherically symmetric solutions can be found by requiring that the functions $A_i,~i=1,2,3$, $\psi$ and $\chi$ are functions of the *r* coordinate only.

### Static spherically symmetric affine symmetry

We have here $\textbf{X}=\frac{1}{C_0}\partial _t$ as the affine static spherically symmetric generator. The coframe components are now:

45 $$\begin{aligned} A_1=A_1\left( r\right) ,\quad \quad A_2=A_2\left( r\right) ,\quad \quad A_3=A_3\left( r\right) , \end{aligned}$$

and $\chi =\chi (r), \psi =\psi (r)$. We still have the coordinate freedom to choose $A_3(r)=r$, which we will exploit later.

For the antisymmetric static FEs, we will use the following version of Eq. (19) as follows:

46 $$\begin{aligned} h^a_{\;\;\mu }=Diag\left[ A_1\left( r\right) , A_2\left( r\right) , A_3\left( r\right) , A_3\left( r\right) \,\sin \theta \right] . \end{aligned}$$

The functions parameterizing the spin-connection described by Eq. (21) become:

47 $$\begin{aligned} W_1 \left( r\right)&= 0 , \ \ \ W_2 \left( r\right) = -\partial _r\,\chi \left( r\right) ,\nonumber \\ W_3 \left( r\right)&= \cosh \left( \psi \left( r\right) \right) \cos \left( \chi \left( r\right) \right) ,\nonumber \\ W_4 \left( r\right)&= \cosh \left( \psi \left( r\right) \right) \sin \left( \chi \left( r\right) \right) , \nonumber \\ W_5 \left( r\right)&= 0 ,\nonumber \\ W_6 \left( r\right)&= -\partial _r\,\psi \left( r\right) ,\nonumber \\ W_7 \left( r\right)&= \sinh \left( \psi \left( r\right) \right) \cos \left( \chi \left( r\right) \right) ,\nonumber \\ W_8 \left( r\right)&= \sinh \left( \psi \left( r\right) \right) \sin \left( \chi \left( r\right) \right) . \end{aligned}$$

### Antisymmetric FEs

Further, we assume that the source is a perfect fluid having energy density $\rho =\rho (r)$ and pressure $P=P(r)$ with comoving fluid velocity

$$\begin{aligned} \textbf{u} = \frac{1}{A_1}\partial _t. \end{aligned}$$

For the antisymmetric FEs, the equivalent of Eqs. (22a) and (22b) are described by the following relations:

48 $$\begin{aligned} 0= & {} \frac{F''\left( T\right) \,\partial _r\,T}{\kappa \,A_2\,A_3}\,\left[ \cos \,\chi \,\sinh \,\psi \right] \quad \text {and}\nonumber \\ 0= & {} \frac{F''\left( T\right) \,\partial _r\,T}{\kappa \,A_2\,A_3}\,\left[ \sin \,\chi \,\cosh \,\psi \right] . \end{aligned}$$

The Eq. (48) admits the solution (for $T \ne \text {const}$):

- $\sin \,\chi =0$ and $\sinh \psi =0$: $\chi = n\,\pi$ and $\psi =0$ where $n \in \mathbb {Z}$ is an integer and $\cos \,\chi =\cos \left( n\,\pi \right) =\pm 1 = \delta$ and $\cosh \psi =1$.

It is curious to note that in this case ($T\not = 0$), most of the spin connection coefficients become zero. The only non-trivial spin connection components are

49 $$\begin{aligned} \omega _{233}=\omega _{244}=\frac{\delta }{A_3}, \text {\ and\ } \omega _{344}=-\frac{\cos (\theta )}{A_3\sin (\theta )}. \end{aligned}$$

This, of course, is in agreement with what is already known in the literature [8, 34]. The torsion scalar becomes

50 $$\begin{aligned} T=-2\left( \frac{\delta }{A_3}+\frac{\partial _r A_3}{A_2 A_3}\right) \left( \frac{\delta }{A_3}+\frac{\partial _r A_3}{A_2A_3}+2\frac{\partial _r A_1}{A_2A_1}\right) , \end{aligned}$$

and we note that the “axial” part of the torsion scalar is identically zero [34].

### Symmetric FEs

The full set of Eqs. (24a)–(24c) above (using the restrictions obtained from the antisymmetric FE) then becomes (removing all time dependencies):

51a $$\begin{aligned}&\kappa \rho =-\frac{1}{2}\left( F(T)-TF'(T)\right) -2F''(T)\left( \frac{\partial _r T}{A_2}\right) \left( \frac{\delta }{A_3}+\frac{\partial _r A_3}{A_2A_3}\right) \nonumber \\&\quad +F'(T)\left[ \!-2\frac{\partial _r^2 A_3}{A_2^2A_3}+2\left( \frac{\partial _r A_2}{A_2^2}\right) \left( \frac{\partial _r A_3}{A_2 A_3}\right) -\left( \frac{\partial _r A_3}{A_2A_3}\right) ^2+\frac{1}{A_3^2}\!\right] , \end{aligned}$$

51b $$\begin{aligned}&\kappa P=\frac{1}{2}\left( F(T)-TF'(T)\right) \nonumber \\&\quad -F'(T)\left[ \!-2\left( \frac{\partial _r A_1}{A_2A_1}\right) \left( \frac{\partial _r A_3}{A_2 A_3}\right) -\left( \frac{\partial _r A_3}{A_2A_3}\right) ^2+\frac{1}{A_3^2}\!\right] , \end{aligned}$$

51c $$\begin{aligned}&\frac{\kappa }{2} (\rho +3P)=\frac{1}{2}\left( F(T)-TF'(T)\right) +F''(T)\left( \frac{\partial _r T}{A_2}\right) \left( \frac{\partial _r A_1}{A_2A_1}\right) \nonumber \\&\quad +F'(T)\left[ \frac{\partial _r^2 A_1}{A_2^2A_1}-\left( \frac{\partial _r A_1}{A_2A_1}\right) \left( \frac{\partial _r A_2}{A_2A_2}\right) +2\left( \frac{\partial _r A_1}{A_2A_1}\right) \left( \frac{\partial _r A_3}{A_2 A_3}\right) \right] . \end{aligned}$$

The eqns. above can be supplemented with the energy–momentum conservation eqn.

52 $$\begin{aligned} (\rho +P)\partial _rA_1+A_1\partial _r P = 0, \end{aligned}$$

which follows from Eqs. (51a)–(51c). In the case of TEGR, where $F(T)=T$, the right-hand side of the first line in each of Eqs. (51a)–(51c) above is trivial and only the second line in each eqn. remains.

We note that Eqs. (51a)–(51c) constitute three FEs (instead of four for the symmetric parts, because the component (1,2) becomes trivial without the time dependence) for $A_j\left( r\right)$, $\rho (r), P(r)$ and $F\left( T\left( r\right) \right)$. We are now able to solve these FEs for a given $F\left( T\left( r\right) \right)$, using an eqn. of state between $\rho$ and *P* and using the final coordinate condition.

We could also express the symmetric FEs as [see Eqs. (24a)–(24c)]:

53a $$\begin{aligned}{} & {} 0 = -F''\left( T\right) \,\left( \partial _r\,T\right) \,k_1\left( r\right) +F'\left( T\right) \,g_1\left( r\right) , \end{aligned}$$

53b $$\begin{aligned}{} & {} \kappa \,[\rho +P] = -2\,F''\left( T\right) \,\left( \partial _r\,T\right) \,k_2\left( r\right) +2\,F'\left( T\right) \,g_2\left( r\right) , \nonumber \\ \end{aligned}$$

53c $$\begin{aligned}{} & {} \left[ \kappa \,\rho +\frac{F\left( T\right) }{2}\right] = -2\,F''\left( T\right) \,\left( \partial _r\,T\right) \,k_3\left( r\right) +2\,F'\left( T\right) \,g_3\left( r\right) , \nonumber \\ \end{aligned}$$

and the corresponding conservation equation, where the functions $k_i$ and $g_i$ ($i=1\,-\,3$) are given by:

54a $$\begin{aligned} g_1&= \frac{1}{A_1\,A_2^3\,A_3^2}\Bigg [-A_2\,A_3^2\,\left( \partial _r^2\,A_1\right) -A_1\,A_2\,A_3\, \left( \partial _r^2\,A_3\right) \nonumber \\&\quad +A_1\,A_2\,\left( \partial _r\,A_3\right) ^2+\partial _r\, \left( A_1\,A_2\right) \,A_3\,\left( \partial _r\,A_3\right) \nonumber \\&\quad +A_3^2\left( \partial _r\,A_1\right) \left( \partial _r\,A_2\right) -A_1\,A_2^3\Bigg ] , \end{aligned}$$

54b $$\begin{aligned} g_2&= \frac{1}{A_1\,A_2^3\,A_3}\Bigg [-A_1\,A_2\,\left( \partial _r^2\,A_3\right) +\partial _r\, \left( A_1\,A_2\right) \,\left( \partial _r\,A_3\right) \Bigg ] , \end{aligned}$$

54c $$\begin{aligned} g_3&= \frac{1}{A_1\,A_2^3\,A_3^2}\Bigg [-A_1\,A_2\,A_3\,\left( \partial _r^2\,A_3\right) -A_1\,A_2\,\left( \partial _r\,A_3\right) ^2\nonumber \\&\quad -A_2\,A_3\left( \partial _r\,A_3\right) \left( \partial _r\,A_1\right) -\delta \,A_1\,A_2^2\,\left( \partial _r\,A_3\right) \nonumber \\&\quad +A_1\,A_3\,\left( \partial _r\,A_2\right) \left( \partial _r\,A_3\right) -\delta \,A_2^2\,A_3\left( \partial _r\,A_1\right) \Bigg ] , \end{aligned}$$

54d $$\begin{aligned} k_1&= \frac{1}{A_1\,A_2^3\,A_3^2}\bigg [A_1\,A_2\,A_3\,\left( \partial _r\,A_3\right) +A_2\,A_3^2 \left( \partial _r\,A_1\right) \nonumber \\&\quad +\delta \,A_1\,A_2^2\,A_3\bigg ], \end{aligned}$$

54e $$\begin{aligned} k_2&= k_3 = \frac{1}{A_2^2\,A_3}\left[ \left( \partial _r\,A_3\right) +\delta \,A_2\right] . \end{aligned}$$

It is possible to rearrange the symmetric part of the FEs from Eq. (53a) to get:

55 $$\begin{aligned} F''(T(r))\left( \partial _r T(r)\right) \,k_1(r)=F'(T(r))\,g_1(r), \end{aligned}$$

where $k_1$ and $g_1$ are given by Eqs. (54a) and (54d). We can write the Eq. (55) as:

56 $$\begin{aligned} \partial _r\,\left[ \ln \,F'\left( T(r)\right) \right] =\frac{g_1\left( r\right) }{k_1\left( r\right) }, \end{aligned}$$

and obtain integral representations for $F'(T(r))$.

We could look for solutions for a particular *F*(*T*) or for a specific eqn. of state; for example, if we assume that $P=\alpha \rho$, after a redefinition of the spatial derivative an explicit integration is possible.

### Vacuum solutions

Let us consider Eqs. (53a)–(53c) in the special case $P\left( r\right) =0$ and $\rho \left( r\right) =0$. The antisymmetric FEs are expressed by Eqs. (48) and we hence set $\sin \chi = 0$, $\cos \chi = \delta$ and $\psi =0$. Equations (53a)–(53c) can be written:

57a $$\begin{aligned}{} & {} F''\left( T\right) \,\left( \partial _r\,T\right) \,k_1\left( r\right) = F'\left( T\right) \,g_1\left( r\right) , \end{aligned}$$

57b $$\begin{aligned}{} & {} F''\left( T\right) \,\left( \partial _r\,T\right) \,k_2\left( r\right) = F'\left( T\right) \,g_2\left( r\right) , \end{aligned}$$

57c $$\begin{aligned}{} & {} \frac{F\left( T\right) }{4} = -F''\left( T\right) \,\left( \partial _r\,T\right) \,k_3\left( r\right) +F'\left( T\right) \,g_3\left( r\right) , \nonumber \\ \end{aligned}$$

where

58a $$\begin{aligned} g_1&=\frac{1}{A_1\,A_2^3\,A_3^2}\Bigg [-A_2\,A_3^2\,\left( \partial _r^2\,A_1\right) -A_1\,A_2\,A_3\,\left( \partial _r^2\,A_3\right) \nonumber \\&\quad +A_1\,A_2\,\left( \partial _r\,A_3\right) ^2 +\partial _r\,\left( A_1\,A_2\right) \,A_3\,\left( \partial _r\,A_3\right) \nonumber \\&\quad +A_3^2\left( \partial _r\,A_1\right) \left( \partial _r\,A_2\right) -A_1\,A_2^3\Bigg ], \end{aligned}$$

58b $$\begin{aligned} g_2&= \frac{1}{A_1\,A_2^3\,A_3}\Bigg [-A_1\,A_2\,\left( \partial _r^2\,A_3\right) +\partial _r\, \left( A_1\,A_2\right) \,\left( \partial _r\,A_3\right) \Bigg ], \end{aligned}$$

58c $$\begin{aligned} g_3&= \frac{1}{A_1\,A_2^3\,A_3^2}\Bigg [-A_1\,A_2\,A_3\,\left( \partial _r^2\,A_3\right) -A_1\,A_2\, \left( \partial _r\,A_3\right) ^2\nonumber \\&\quad -A_2\,A_3\left( \partial _r\,A_3\right) \left( \partial _r\,A_1\right) -\delta \,A_1\,A_2^2\,\left( \partial _r\,A_3\right) \nonumber \\&\quad +A_1\,A_3\,\left( \partial _r\,A_2\right) \left( \partial _r\,A_3\right) -\delta \,A_2^2\,A_3\left( \partial _r\,A_1\right) \Bigg ], \end{aligned}$$

58d $$\begin{aligned} k_1&= \frac{1}{A_1\,A_2^3\,A_3^2}\Bigg [A_1\,A_2\,A_3\,\left[ \delta \,A_2+\left( \partial _r\,A_3\right) \right] +A_2\,A_3^2\left( \partial _r\,A_1\right) \Bigg ], \end{aligned}$$

58e $$\begin{aligned} k_2&=k_3 = \frac{1}{A_2^2\,A_3}\left[ \delta \,A_2+\left( \partial _r\,A_3\right) \right] . \end{aligned}$$

From the Eqs. (57a) and (57b) we obtain the relation $g_1\,k_2=g_2\,k_1$:

59 $$\begin{aligned}&\Bigg [-A_2\,A_3^2\,\left( \partial _r^2\,A_1\right) -A_1\,A_2\,A_3\,\left( \partial _r^2\,A_3\right) +A_1\,A_2\,\left( \partial _r\,A_3\right) ^2\nonumber \\&\qquad +\partial _r\,\left( A_1\,A_2\right) \,A_3\,\left( \partial _r\,A_3\right) \nonumber \\&\qquad +A_3^2\left( \partial _r\,A_1\right) \left( \partial _r\,A_2\right) -A_1\,A_2^3\Bigg ]\, \left[ A_1\,A_2\,\left( \partial _r\,A_3\right) +\delta \,A_1\,A_2^2\right] \nonumber \\&\quad = \Bigg [-A_1\,A_2\,\left( \partial _r^2\,A_3\right) +\partial _r\,\left( A_1\,A_2\right) \, \left( \partial _r\,A_3\right) \Bigg ]\,\nonumber \\&\qquad \times \left[ A_1\,A_2\,A_3\,\left( \partial _r\,A_3\right) +A_2\,A_3^2 \left( \partial _r\,A_1\right) +\delta \,A_1\,A_2^2\,A_3\right] \end{aligned}$$

and from (57c), using (57b), the expression

60 $$\begin{aligned}&\frac{{k_1\left( r\right) }}{4\left[ {k_1\left( r\right) } g_3\left( r\right) - {g_1\left( r\right) \,k_3\left( r\right) }\right] } = \frac{d}{dT}\left( \ln F(T)\right) , \end{aligned}$$

which leads to an integral representations of the solutions, where $T\left( r\right)$ is given by Eq. (50), which further simplifies using the Eq. (57c).

It is of interest to consider the teleparallel version of *Minkowski* vacuum, in which additional conditions are imposed, and there are constraints on the form of the arbitary function *F*(*T*) when *T* vanishes. Perturbations of teleparallel *Minkowski* vacuum was studied in [47].

#### Specific coordinate choice

Redefining the *r* coordinate may simplify the governing eqns. further. We shall redefine *r* to set $A_3\left( r\right) =r$ below. In this case, Eqs. (53a)–(53c) have the components:

61a $$\begin{aligned} g_1&=\frac{1}{A_1\,A_2^3\,r^2}\Bigg [-A_2\,r^2\,\left( \partial _r^2\,A_1\right) +A_1\,A_2\nonumber \\&\quad +\partial _r\, \left( A_1\,A_2\right) \,r+r^2\left( \partial _r\,A_1\right) \left( \partial _r\,A_2\right) -A_1\,A_2^3\Bigg ] , \end{aligned}$$

61b $$\begin{aligned} g_2&= \frac{1}{A_1\,A_2^3\,r}\Bigg [\partial _r\,\left( A_1\,A_2\right) \Bigg ] , \end{aligned}$$

61c $$\begin{aligned} g_3&= \frac{1}{A_1\,A_2^3\,r^2}\Bigg [-A_1\,A_2-A_2\,r\left( \partial _r\,A_1\right) -\delta \,A_1\,A_2^2\nonumber \\&\quad +A_1\,r\,\left( \partial _r\,A_2\right) -\delta \,A_2^2\,r\left( \partial _r\,A_1\right) \Bigg ], \end{aligned}$$

61d $$\begin{aligned} k_1&= \frac{1}{A_1\,A_2^3\,r^2}\left[ A_1\,A_2\,r+A_2\,r^2\left( \partial _r\,A_1\right) +\delta \,A_1\,A_2^2\,r\right] , \end{aligned}$$

61e $$\begin{aligned} k_2&= k_3 = \frac{1}{A_2^2\,r}\left[ 1+\delta \,A_2\right] . \end{aligned}$$

The torsion scalar is expressed as:

62 $$\begin{aligned} T(r)=-2\left( \frac{\delta }{r}+\frac{1}{A_2\,r}\right) \left( \frac{\delta }{r}+\frac{1}{A_2\,r}+2\frac{\partial _r A_1}{A_2\,A_1}\right) . \end{aligned}$$

This can only be done in the general case $A_3 \ne$ constant. There are restrictions on constraining $A_1$, since the conservation eqn. relates its radial derivative with $\partial _r P = 0$. An alternative option might be to set $A_2 = 1/A_1$.

#### Schwarzschild-like solutions

Let us consider the coframe Eq. (46) in which $A_2\left( r\right) =\frac{1}{A_1\left( r\right) }$ and $A_3\left( r\right) =r$, corresponding to *Schwarzschild*-like solutions. Equation (48) yield $\sin \,\chi =0$ and $\sinh \psi =0$: $\cos \,\chi =\cos \left( n\,\pi \right) =\pm 1 = \delta$ and $\cosh \psi =1$. The three non-trivial symmetric FEs are expressed as in Eqs. (53a)–(53c) with the components:

63a $$\begin{aligned} g_1 =&\frac{A_1^2}{r^2}\Bigg [-A_1^{-1}\,r^2\,\left( \partial _r^2\,A_1\right) +1-r^2\,A_1^{-2}\left( \partial _r\,A_1\right) ^2-A_1^{-2}\Bigg ] , \end{aligned}$$

63b $$\begin{aligned} g_2 =&0 , \end{aligned}$$

63c $$\begin{aligned} g_3 =&\frac{A_1^2}{r^2}\Bigg [-1-2\,A_1^{-1}\,r\left( \partial _r\,A_1\right) -\delta \,A_1^{-1} -\delta \,A_1^{-2}\,r\left( \partial _r\,A_1\right) \Bigg ] , \end{aligned}$$

63d $$\begin{aligned} k_1 =&\frac{A_1^2}{r^2}\left[ r+A_1^{-1}\,r^2\left( \partial _r\,A_1\right) +\delta \,A_1^{-1}\,r\right] , \end{aligned}$$

63e $$\begin{aligned} k_2=&\, k_3 = \frac{A_1^2}{r}\left[ 1+\delta \,A_1^{-1}\right] . \end{aligned}$$

Finally, from Eq. (50) we obtain the torsion scalar:

64 $$\begin{aligned} T(r)=-4\delta \left[ \frac{\frac{dA_1}{dr}}{r}+\frac{A_1}{r^2}\right] -\frac{2}{r^2} -\frac{4\,A_1\,\frac{dA_1}{dr}}{r}-\frac{2\,A_1^2}{r^2}. \nonumber \\ \end{aligned}$$

The solutions of the Eqs. (53a)–(53c) for Eqs. (63a)–(63e) include the Schwarzschild spacetime ($A_1(r)=\sqrt{1-\frac{r_S}{r}}$) as well as the Reissner–Nordstrom spacetime ($A_1(r)=\sqrt{1-\frac{r_S}{r} +\frac{r_Q^2}{r^2}}$), as examples. We note that for $\rho =P=0$ we have that $F''=0$ or $T=$ constant, which leads to the GR case with a cosmological constant.

Note that the Birkhoff theorem does not generally hold in *F*(*T*) theories. In addition, a perturbed solution around Minkowski spacetime and solar system constraints on the parameters in *F*(*T*) gravity were first discussed in [17, 18].

### Analysis and power-law solutions

We summarize the governing eqns. in the vacuum case with $A_3=r$ (we also could consider the quadratic function *F*). The FEs (57a)–(57c) with components (61a)–(61e) are given by:

65a $$\begin{aligned} 0= & {} g_1k_2-g_2k_1, \end{aligned}$$

65b $$\begin{aligned} g_2= & {} k_2 \partial _r\,\left[ \ln \,F'\left( T(r)\right) \right] , \end{aligned}$$

65c $$\begin{aligned} \frac{F(T)}{4}= & {} F'(T)\left( g_3-g_2\right) . \end{aligned}$$

or in terms of the original variables we have explicitly

66a $$\begin{aligned} 0 =&\left( \delta \,A_2+1\right) \left( \frac{\partial _{r}^2 A_1}{A_1}\right) +\left( \frac{\partial _r A_1}{A_1}\right) ^2-\delta \left( \frac{\partial _r A_1}{A_1}\right) \nonumber \\&\quad \times \left( \partial _r A_2\right) +\frac{1}{r^2}\left( \delta \,A_2+1\right) \left( A_2^2-1\right) , \end{aligned}$$

66b $$\begin{aligned} 0 =&\left( \delta \,A_2+1\right) \partial _r\,\left[ \ln \,F'\left( T(r)\right) \right] -\partial _r\left[ \ln \,(A_1 A_2)\right] , \end{aligned}$$

66c $$\begin{aligned} \frac{F(T)}{4} =&\frac{F'(T)}{r^2 A_2^2}\left[ -\left( \delta \,A_2+2\right) \left( \frac{r\,\partial _r A_1}{A_1}\right) -\left( \delta \,A_2+1\right) \right] . \end{aligned}$$

It is noteworthy that the torsion scalar, given explicitly earlier, can be written as

67 $$\begin{aligned} T(r)=2A_2^2\,(k_2^2-2k_1 k_2). \end{aligned}$$

We already know that solutions with $A_1\,A_2=$ constant lead to GR-like solutions. We can seek power-law solutions in general. The only case in which we cannot set $A_3=r$ is when $A_3=$ constant, which we must treat separately. Finally, we consider the case $A_2=$ constant, in which a number of new solutions can be obtained.

#### Power-law solutions

In general, for $A_1(r)=a_0\,r^a$ and $A_2(r)=b_0\,r^b$, after setting $A_3=r$ as a coordinate choice, we get from Eqs. (62) and (66a)–(66c):

68a $$\begin{aligned}&0 = \frac{1}{b_0^2\,r^{2b}}\left[ \delta \,\left( a^2-a(1+b)-1\right) +\frac{\left( 2a^2-a-1\right) }{b_0\,r^{b}}\right] \nonumber \\&\qquad +\left[ \frac{1}{b_0\,r^{b}}+\delta \right] , \end{aligned}$$

68b $$\begin{aligned}&F'(T(r)) = F_1\,\exp \left[ (a+b)\int \,\frac{dr}{r\,\left( \delta \,b_0\,r^b+1\right) } \right] , \end{aligned}$$

68c $$\begin{aligned}&F'(T(r)) = -\frac{b_0\,r^{b+2}\,F(T(r))}{4\left[ \left( \delta +\frac{2}{b_0\,r^{b}}\right) \,a +\left( \delta +\frac{1}{b_0\,r^{b}}\right) \right] } , \end{aligned}$$

68d $$\begin{aligned}&T(r)= -\frac{2}{r^2}\left[ \left( \delta +\frac{1}{b_0\,r^{b}}\right) ^2+\frac{2\,a}{b_0\,r^{b}}\, \left( \delta +\frac{1}{b_0\,r^{b}}\right) \right] . \end{aligned}$$

The only possible solutions for Eq. (68a) is obtained by setting $b=0$, which means we reduce to a special case of the general case in Eq. (73) with $y_1=0$ below.

#### Solution for $A_3(r)=$ constant

With $A_3=c_0$ and the coordinate choice $A_2=b_0=1$, using Eqs. (50) and (54a)–(54e) we obtain:

69a $$\begin{aligned} A_1''+\frac{A_1}{c_0^2}=&0 , \end{aligned}$$

69b $$\begin{aligned} \partial _r\left[ \ln F'(T)\right] =&0 , \end{aligned}$$

69c $$\begin{aligned} \partial _r\left[ \ln F(T)\right] =&-\frac{\delta \,c_0\,A_1\,T'(r)}{4\,A_1'} , \end{aligned}$$

69d $$\begin{aligned} T(r)=&-\frac{2}{c_0^2}-\frac{4\delta \,A_1'}{c_0\,A_1} . \end{aligned}$$

From Eq. (69b), we find that $F'(T)=F_{1}=$ constant, leading to $F(T)=F_{1} \,T+ F_{0}$. From Eq. (69d), we find that $\left( T(r)+\frac{2}{c_0^2}\right) =-\frac{4A_1'}{\delta \,c_0\,A_1}$ and then Eq. (69c) becomes:

70 $$\begin{aligned} F(T)=F_1\left[ T+\frac{2}{c_0^2}\right] . \end{aligned}$$

Since Eq. (69a) leads to an oscillating $A_1(r)$ of frequency $\omega _0=\frac{1}{c_0}$, it is not physically relevant.

### Solutions for $A_3(r)=r$ and $A_2=$ constant

We consider solutions for *F*(*T*(*r*)) by using Eqs. (61a)–(61e) as $g_i$ and $k_i$ components in Eqs. (65a)–(65c), leading to the FEs described by Eqs. (54a)–(54e). By setting $A_2(r)=b_0=$ constant in Eqs. (54a)–(54e), this assumption leads to the simplified FEs:

71a $$\begin{aligned}&0= \left[ \frac{A_1''}{A_1}+\frac{1}{r^2}\,\left( b_0^2-1\right) \right] \left( \delta \,b_0+1\right) +\left( \frac{A_1'}{A_1}\right) ^2=0 , \end{aligned}$$

71b $$\begin{aligned}&F'(T(r)) = F_1\,A_1^{\frac{1}{\left( \delta \,b_0+1\right) }} , \end{aligned}$$

71c $$\begin{aligned}&F'(T(r)) = s-\frac{b_0^2\,r^2\,F(T(r))}{4\left[ \left( \delta \,b_0+2\right) \, \left( \frac{r\,A_1'}{A_1}\right) +\left( \delta \,b_0+1\right) \right] }, \end{aligned}$$

where $F_1$ is an integration constant and $\delta b_0 \ne -1$. By setting $y(r)=\ln \,\left( A_1(r)\right)$, Eq. (71a) becomes a non-linear ODE:

72 $$\begin{aligned} 0 =&\left( \delta \,b_0+1\right) \,y''+\left( \delta \,b_0+2\right) \,y'^2+\, \frac{\left( \delta \,b_0-1\right) \left( \delta \,b_0+1\right) ^2}{r^2}. \end{aligned}$$

We will solve Eq. (72) in the general case and then we will consider the special values of $\delta \,b_0=+1$ and $\delta \,b_0=-2$. Note, if $\delta \,b_0=-1$, then the torsion scalar is identically zero (see Eq. (50)) and is not of interest.

#### General solution

The general solution of Eq. (72) is ($\delta \,b_0\ne -2$ and $\delta \,b_0\ne \pm 1$):

73 $$\begin{aligned} y(r)&= y_2+\frac{(\delta \,b_0+1)}{2(\delta \,b_0+2)}(1-S)\,\ln \,r \nonumber \\&\quad +\frac{(\delta \,b_0+1)}{(\delta \,b_0+2)}\ln \left[ r^{S}+y_1\right] , \end{aligned}$$

where $y_1$ and $y_2$ are arbitrary constants and

74 $$\begin{aligned} S=\pm \sqrt{1-4\left( \delta \,b_0-1\right) \left( \delta \,b_0+2\right) }. \end{aligned}$$

If $y_1=0$, then all solutions to Eq. (73) yield power-law solutions for $A_1(r)$. By taking the exponential of Eq. (73), we obtain:

75 $$\begin{aligned} A_1(r)= a_0\,r^{\frac{\left( \delta \,b_0+1\right) }{2\,\left( \delta \,b_0+2\right) } \left( 1+S\right) }\,\left( 1+y_1\,r^{-S}\right) ^{\frac{\left( \delta \,b_0+1\right) }{\left( \delta \,b_0+2\right) }} , \end{aligned}$$

where $a_{0}$ is a constant. Equations (71b) and (71c) become:

76a $$\begin{aligned} F'(T(r))&= F_2\,r^{\frac{\left( 1+S\right) }{2\,\left( \delta \,b_0+2\right) }}\, \left( 1+y_1\,r^{-S}\right) ^{\frac{1}{\left( \delta \,b_0+2\right) }} , \end{aligned}$$

76b $$\begin{aligned} F'(T(r))&=-\frac{b_0^2\,r^2\,F(T)}{2\left( \delta \,b_0+1\right) \left( 3-S\right) \left[ 1+\frac{2S}{\left( 3-S\right) \left[ 1+y_1\,r^{-S}\right] }\right] } , \end{aligned}$$

where $S\ne 3$ and $F_2=F_1\,a_0^{\frac{1}{\left( \delta \,b_0+1\right) }}$. The Eq. (50) for the torsion scalar becomes:

77 $$\begin{aligned} T(r)&= -\frac{2\left( \delta b_0+1\right) ^2\,\left( 3+\delta \,b_0-S\right) }{b_0^2\,(\delta \,b_0+2)\,r^2}\nonumber \\&\quad \times \left[ 1+\frac{2S}{\left( 3+\delta \,b_0-S\right) \left[ 1+y_1\,r^{-S}\right] }\right] ,\nonumber \\&= \frac{T_0}{r^2}\,\left[ 1+\frac{T_1}{\left[ 1+y_1\,r^{-S}\right] }\right] , \end{aligned}$$

where $T_0$ and $T_1$ are constants depending on $b_0$ and *S*. By combining Eqs. (76a) and (76b), we obtain *F*(*T*):

78 $$\begin{aligned} F(T(r))&=-\frac{2\left( \delta \,b_0+1\right) \left( 3-S\right) }{b_0^2}\nonumber \\&\quad \times F_2\,\left[ 1 +F_3\,\left( 1+y_1\,r^{-S}\right) ^{-1}\right] \,\nonumber \\&\quad \times \left( 1+y_1\,r^{-S}\right) ^{\frac{1}{\left( \delta \,b_0+2\right) }}\,r^{\frac{\left( 1+S\right) }{2\,\left( \delta \,b_0+2\right) }-2}, \end{aligned}$$

where $F_3=\frac{2S}{\left( 3-S\right) }$ (for $S \ne 3$). Equation (78) is the general expression for *F*(*T*(*r*)) and is a new result. We consider the following cases:

1. $\mathbf{y_1=0}$ (Power-law solutions for *F*(*T*)): From Eqs. (77) and (78) for all *S* values, we find:
  79a $$\begin{aligned} T(r)&= \frac{T_0\left( 1+T_1\right) }{r^2} , \end{aligned}$$
  79b $$\begin{aligned} F(T)&=-\frac{2\left( \delta \,b_0+1\right) \left( 3-S\right) }{b_0^2}\,F_2\,\left( 1+F_3\right) \nonumber \\&\quad \times \left( \frac{T}{T_0\left( 1+T_1\right) }\right) ^{1-\frac{\left( 1+S\right) }{4\,\left( \delta \,b_0+2\right) }}. \end{aligned}$$
  We obtain from Eqs. (79a) and (79b) the power-law solution of Golovnev–Guzman [22].
2. $$\mathbf{y_1 \ne 0}$$: Equation (77) becomes:
  80 $$\begin{aligned} \left( \frac{T}{T_0}\right) \left( r^2+y_1\,r^{2-S}\right) -y_1\,r^{-S}-\left( 1+T_1\right) =0. \end{aligned}$$
  To find *F*(*T*) explicitly, we need to find *r* as a function of *T* in Eq. (80). There are some solvable cases for specific values of *S*:
  - $\mathbf{S=0}$: We obtain that $\delta \,b_0=\frac{\pm \sqrt{10}-1}{2}$ from Eq. (74), $T_0 =-\frac{2}{27}\left( 25\pm 34\sqrt{10}\right)$, $T_1=0$, $F_2=F_1\,a_0^{\frac{2}{9}\left( -1\pm \sqrt{10}\right) }$ and $F_3=0$. Equation (80) leads to:
    81 $$\begin{aligned} r(T)=\left( \frac{T}{T_0}\right) ^{-\frac{1}{2}}. \end{aligned}$$
    Then Eq. (78) will be:
    82 $$\begin{aligned} F(T)&= -\frac{4}{27}\,\left( 31\pm 13\sqrt{10}\right) F_2\,\left( 1+y_1\right) ^{\frac{2}{\left( 3\pm \sqrt{10}\right) }}\nonumber \\&\quad \times \left( \frac{T}{T_0}\right) ^{1-\frac{1}{2\left( 3\pm \sqrt{10}\right) }}, \end{aligned}$$
    which is again a power-law solution for *F*(*T*) [22].
  - $\mathbf{S=\pm 1}$: We obtain $\delta \,b_0=1$ or $-2$ from Eq. (74), both special cases. We consider these specific cases below.
  - $\mathbf{S=2}$: We obtain $\delta \,b_0=\frac{\pm \sqrt{6}-1}{2}$ from Eq. (74). We find that $T_0=-\frac{2}{75} \left( 117\pm 62\sqrt{6}\right)$, $T_1=-\frac{8}{5}\left( 1\mp \sqrt{6}\right)$, $F_2=F_1\,a_0^{\frac{2}{5}\left( -1\pm \sqrt{6}\right) }$ and $F_3=4$. Equation (80) leads to:
    83 $$\begin{aligned} r^2(T)&=\frac{1}{2}\left( \frac{T_0 (1+T_1)}{T}-y_1\right) \nonumber \\&\quad \times \left[ 1\pm \sqrt{1+\frac{4\,y_1\,\frac{T_0}{T}}{\left( \frac{T_0 (1+T_1)}{T}-y_1\right) ^2}} \right] . \end{aligned}$$
    Equation (78) becomes:
    84 $$\begin{aligned}&F(T)= -\frac{4}{25}\,\left( 19\pm 9\sqrt{6}\right) \nonumber \\&F_2\,\frac{\left( 5\,r^2(T)+y_1\right) }{\left( r^2(T)+y_1\right) ^{1-\frac{2}{3\pm \sqrt{6}}}\, \left( r(T)\right) ^{2+\frac{1}{3\pm \sqrt{6}}}}, \end{aligned}$$
    where *r*(*T*) is the Eq. (83). This is a new result.
  - $\mathbf{S=-2}$: For $\delta \,b_0=\frac{\pm \sqrt{6}-1}{2}$ and by using Eq. (74), we get that $T_0=-2\left( 7\pm 2\sqrt{6}\right)$, $T_1=-\frac{8}{75}\left( 9\mp \sqrt{6}\right)$, $F_2=F_1\,a_0^{\frac{2}{5}\left( -1\pm \sqrt{6}\right) }$ and $F_3=-\frac{4}{5}$. Equations (80) leads to:
    85 $$\begin{aligned}&r^2(T) =\frac{1}{2}\left[ \left( \frac{T}{T_0}\right) ^{-1}-\frac{1}{y_1}\right] \nonumber \\&\times \left[ 1\pm \sqrt{1+\frac{4(1+T_1)\left( \frac{T}{T_0}\right) }{y_1\left[ 1-\frac{1}{y_1}\left( \frac{T}{T_0}\right) \right] ^2}} \right] . \end{aligned}$$
    Equation (78) becomes:
    86 $$\begin{aligned}&F(T)= -\frac{4}{5}\,\left( 19\pm 9\sqrt{6}\right) \nonumber \\&\times F_2\,\frac{\left( 1+5y_1\,r^2(T)\right) }{\left( 1+y_1\,r^2(T)\right) ^{1-\frac{2}{3\pm \sqrt{6}}}\, \left( r(T)\right) ^{2+\frac{1}{3\pm \sqrt{6}}}} , \end{aligned}$$
    where *r*(*T*) is the Eq. (85). This is also a new result.
  - **Limits on** $\textbf{S}$: By using Eq. (74), we find that real values for $\delta b_0$ are only possible when $-\sqrt{10}\, \le \, S\, \le \, + \sqrt{10}$. By the extremum test applied to Eq. (74), we find that these occur when $\delta b_0=-\frac{1}{2}$. However we are unable to find *r*(*T*) explicitly from Eq. (77) in this case.
  For the $S=\pm 2$ cases, we obtain new results. For other values of *S*, we obtain a more complex form for the relationship between *T* and *r* in Eq. (80) which might not yield an explicit expression for $r=r(T)$.

#### Special solution: $b_0=-2\delta$

By setting $b_0=-2\delta$, Eq. (72) becomes:

87 $$\begin{aligned} 0 =&y''+\frac{3}{r^2} . \end{aligned}$$

The solution is $y=\, 3\,\ln \,r+y_1\,r+y_2$, which yields

88 $$\begin{aligned} A_1(r)= a_0\,r^3\,e^{y_1\,r}. \end{aligned}$$

Equations (71b) and (71c) then yield:

89 $$\begin{aligned} F(T(r)) = F_2\,r^{-5}\,e^{-y_1\,r} , \end{aligned}$$

where $F_2=\frac{F_1}{a_0}$. Eq. (50) then gives $T(r)= \,\frac{5}{2}\,r^{-2}+y_1\,r^{-1}$, which yields

90 $$\begin{aligned} r^{-1}(T)=\,\frac{1}{5}\left[ -y_1 \pm \sqrt{y_1^2+10\,T}\right] , \end{aligned}$$

and then Eq. (89) yields the new solution:

91 $$\begin{aligned} F(T)&= \frac{F_2}{3125}\,\left[ -y_1 \pm \sqrt{y_1^2+10\,T}\right] ^{5}\nonumber \\&\quad \times \exp \left[ \frac{5y_1}{\left[ y_1 \mp \sqrt{y_1^2+10\,T}\right] }\right] . \end{aligned}$$

For $y_1=0$, Eqs. (88) and (89) become, respectively, $A_1(r)= a_0\,r^3$ and $F(T(r)) = \frac{F_{1}}{a_0}\,r^{-5}$. Then Eq. (90) becomes $r^{-1}(T)=\sqrt{\frac{2}{5}}\,T^{\frac{1}{2}}$ and we find a new power-law solution:

92 $$\begin{aligned} F(T)= \left( \frac{2}{5}\right) ^{\frac{5}{2}}\,F_2\,T^{\frac{5}{2}}. \end{aligned}$$

Note that the case $\delta \,b_0=-2$ in Golovnev–Guzman [22] was treated via a complex ansatz in $A_1(r)$ and using $A_2(r)=\frac{\delta \left( 1-2c_1\,r^2\right) }{c_1\,r^2+1}\,\ne$ constant. Taking the asymptotic limit $r\,\rightarrow \,\infty$ of $A_2(r)$, we obtain that $A_2(r)\,\rightarrow \,-2\delta$. However, Eq. (92) is not in [22] and hence this as a new result.

#### Special solution: $b_0=+\delta$

Equation (72) for $\delta \,b_0=1$ reduces to:

93 $$\begin{aligned} 0 = y''+\frac{3}{2}\,y'^2 . \end{aligned}$$

By integrating, we obtain $y(r)=\frac{2}{3}\,\ln \left( r+y_1\right) +y_2$, which yields

94 $$\begin{aligned} A_1(r)=a_0\,\left( r+y_1\right) ^{\frac{2}{3}}. \end{aligned}$$

Equation (50) then yields:

95 $$\begin{aligned} T(r) = -\frac{8}{r^2}\left( 1+\frac{2r}{3\left( r+y_1\right) }\right) . \end{aligned}$$

From Eq. (95), it is possible to find an explicit expression for *r*(*T*):

96 $$\begin{aligned} r(T) =&-\frac{y_1}{3}+\frac{1}{3T}\Bigg [-\left( y_1\,T\right) ^3+2\sqrt{2}\,T\, \nonumber \\&\times \sqrt{27\,y_1^4\,T^3-312\,y_1^2\,T^2+8000\,T}-48\,y_1\,T^2\Bigg ]^{\frac{1}{3}}\nonumber \\&-\frac{\left( 40-y_1^2\,T\right) }{3}\Bigg [-\left( y_1\,T\right) ^3+2\sqrt{2}\,T\nonumber \\&\times \sqrt{27\,y_1^4\,T^3-312\,y_1^2\,T^2+8000\,T}-48\,y_1\,T^2\Bigg ]^{-\frac{1}{3}}. \end{aligned}$$

By combining Eqs. (71b) and (71c), we obtain the new result:

97 $$\begin{aligned} F(T)=-8\,F_2\,\frac{\left( 2r(T)+y_1 \right) }{\left( r(T)+y_1\right) ^{\frac{2}{3}}\,r^{2}(T)} , \end{aligned}$$

where $$F_2=F_1\,\sqrt{a_0}$$.

For $y_1=0$, Eq. (94) becomes $A_1(r)=a_0\,r^{\frac{2}{3}}$, then Eq. (96) leads to $r(T)=\sqrt{\frac{40}{3}}\,\left( -T\right) ^{-\frac{1}{2}}$. Finally, Eq. (97) becomes

98 $$\begin{aligned} F(T)=-16\,\left( \frac{3}{40}\right) ^{\frac{5}{6}}\,F_2\,\left( -T\right) ^{\frac{5}{6}}, \end{aligned}$$

where $T \le 0$. Eq. (97) is again the power-law solution [22].

In this subsection, we have presented a number of new solutions. We have also recovered the known Golovnev–Guzman power-law solution in a number of special subcases [22].

## Kantowski–Sachs geometry

In order to expand the affine frame symmetry group of the spherically symmetric case to that of a Kantowski–Sachs geometry, we need to consider the following four affine frame symmetry generators:

99 $$\begin{aligned} \textbf{X}_3= & {} \partial _{\phi },~\textbf{X}_2 = - \cos \phi \partial _{\theta } + \frac{\sin \phi }{\tan \theta } \partial _{\phi }, \nonumber \\ \textbf{X}_1= & {} \sin \phi \partial _{\theta } + \frac{\cos \phi }{\tan \theta } \partial _{\phi }, \ \textbf{X}_4 = \partial _r. \end{aligned}$$

The resulting Lie algebra is then

100 $$\begin{aligned}{}[\textbf{X}_1, \textbf{X}_2]= & {} -\textbf{X}_3, [\textbf{X}_1, \textbf{X}_3]\nonumber \\= & {} \textbf{X}_2, [\textbf{X}_2, \textbf{X}_3] = -\textbf{X}_1, [\textbf{X}_{I'}, \mathbf{X_4}] = 0, \end{aligned}$$

where in the last bracket, $I' = 1,2,3$. Using the approach in [16], we find that

101 $$\begin{aligned} \begin{aligned} \mathcal {L}_{\textbf{X}_{I'}} {\textbf{h}}^a&= f_{I'}^{~\hat{i}} \lambda ^a_{\hat{i}~b} {\textbf{h}}^b, \\ \mathcal {L}_{\textbf{X}_4} {\textbf{h}}^a&= 0, \\ \mathcal {L}_{\textbf{X}_I} \omega ^a_{~bc}&= 0, I=1,\ldots 4. \end{aligned} \end{aligned}$$

where

102 $$\begin{aligned} f_I^{\tilde{i}} = \left[ \begin{array}{cccc} \frac{\cos (\phi )}{\sin (\theta )} &{} 0 &{} 0 &{} 0 \\ \frac{\sin (\phi )}{\sin (\theta )} &{} 0 &{} 0 &{} 0 \\ 0 &{} 0 &{} 0 &{} 0 \end{array}\right] \end{aligned}$$

Solving these eqns. leads again to the spherically symmetric ansatz for a spherically symmetric Riemann–Cartan geometry, where now there is only *t*-dependence in the functions of the vierbein and spin-connection described by Eqs. (19) and (20) with $A_i=A_i(t)$ ($i=1,\ldots ,\,3$) and $W_j=W_j(t)$ ($j=1,\ldots \,8$). Requiring that the curvature vanishes then implies that the arbitrary functions described by Eqs. (21) must take the form:

103 $$\begin{aligned} \begin{aligned} W_1&= -\frac{\partial _t \chi }{A_1},\quad W_2 = 0,\quad W_3 = \frac{\cosh (\psi )\cos (\chi )}{A_3}, \\ W_4&= \frac{\cosh (\psi )\sin (\chi )}{A_3},\\ W_5&= -\frac{\partial _t \psi }{A_1}, \quad W_6 = 0, \quad W_7 = \frac{\sinh (\psi ) \cos (\chi )}{A_3}, \\ W_8&= \frac{\sinh (\psi ) \sin (\chi )}{A_3}, \end{aligned} \end{aligned}$$

where $\chi$ and $\psi$ are arbitrary functions of the coordinate *t*.

Without loss of generality, the *t*-coordinate can be rescaled to set $A_1 = 1$, giving the vierbein:

104 $$\begin{aligned} h^a_{~\mu } = \left[ \begin{array}{cccc} 1 &{} 0 &{} 0 &{} 0 \\ 0 &{} A_2(t) &{} 0 &{} 0 \\ 0 &{} 0 &{} A_3(t) &{} 0 \\ 0 &{} 0 &{} 0 &{} A_3(t) \sin (\theta ) \end{array}\right] . \end{aligned}$$

Denoting derivatives with respect to *t* with a prime, $f_{,t} = f'$, the vector-part and axial-part of the torsion are

105 $$\begin{aligned} \begin{aligned} \textbf{V}&= \frac{2A_2 \sinh (\psi ) \cos (\chi )+A_{2}'A_3 + 2A_{3}'A_2)}{A_2 A_3} {\textbf{h}}^1\\&\quad - \frac{2\cosh (\psi ) \cos (\chi ) - \psi ' A_3}{A_3} {\textbf{h}}^2, \\ \textbf{A}&= \frac{4 \cosh (\psi )\sin (\chi )}{3A_3} {\textbf{h}}^1 - \frac{2(\chi ' A_3 - 2\sinh (\psi ) \sin (\chi ))}{3A_3} {\textbf{h}}^2, \end{aligned} \end{aligned}$$

while the tensor-part of the torsion,

$$\begin{aligned} t_{(ab)c} = \hat{T}_{abc} + \hat{T}_{bac}, \end{aligned}$$

where the $\hat{T}_{a[bc]}$ have components:

106 $$\begin{aligned} \begin{aligned} \hat{T}_{112}&= \hat{T}_{332} = \hat{T}_{442} = \frac{-2(\cosh (\psi )\cos (\chi )+\psi ' A_3)}{3 A_3}, \\ \hat{T}_{134}&= \hat{T}_{314} = -\hat{T}_{413} = \frac{-2(\sinh (\psi )\sin (\chi )+\chi ' A_3)}{3 A_3}, \\ \hat{T}_{212}&= \hat{T}_{331} = \hat{T}_{441} \\&= -\frac{2(\sinh (\psi )\cos (\chi )A_2 + A_{3}' A_2 - A_{2}' A_3)}{3A_2 A_3}, \\ \hat{T}_{234}&= \hat{T}_{324} = \hat{T}_{432} = -\frac{\sinh (\psi )\sin (\chi )}{3A_3}. \end{aligned} \end{aligned}$$

We remark that the Kantowski–Sach solutions admitting a $G_6$ in GR, with functions $A_2 = e^{2\sqrt{\Lambda } t}$ and $A_3 = \frac{1}{\Lambda }$ for $\Lambda \in \mathbb {R}$, can at most admit a $G_5$, which arises when $\chi = \pi /2$ and $\psi = 0$. This is due to the fact that the boost and spin isotropy in this solution necessarily requires that all irreducible parts of the torsion tensor vanish.

The antisymmetric part of the *F*(*T*) FEs give

107a $$\begin{aligned} \frac{F_{TT}(T)\, T' \cosh (\psi ) \cos (\chi )}{A_3}&= 0, \end{aligned}$$

107b $$\begin{aligned} \frac{F_{TT}(T)\,T' \sinh (\psi ) \sin (\chi )}{A_3}&=0. \end{aligned}$$

These eqns. imply that $\psi = 0$ and $\chi = \pi /2$ (or *T* is constant).

### Constant torsion scalar

108 $$\begin{aligned} \begin{aligned} T&= -\frac{4(2 (\sinh (\psi ) \cos (\chi )A_3 A_2)' -2 A_{2}' A_{3}'A_3 - {A_{3}'}^2 A_2 + A_2 )}{A_3^2 A_2}. \end{aligned} \nonumber \\ \end{aligned}$$

The algebraically independent components of the symmetric part of the FEs are then

109a $$\begin{aligned} \kappa \rho + \frac{F(T)}{2}&= \left( \frac{4 (\sinh (\psi ) \cos (\chi )A_3 A_2)' + 4 {A_{3}'}^2 A_2 + 8 A_{3}' A_{2}' A_3}{2 A_3^2 A_2} \right) \nonumber \\&\quad \times F_T(T), \end{aligned}$$

109b $$\begin{aligned} -\kappa (\rho + P)&= -\left( \frac{2(A_{3}'' A_2 - A_{3}' A_{2}')}{A_3 A_2} \right) F_T(T), \end{aligned}$$

109c $$\begin{aligned} 0&= \left( \frac{{A_{3}'}^2 A_2 - A_{3}' A_{2}' A_3 + A_2 A_3 A_{3}'' - A_{2}'' A_3^2 + A_2}{A_3^2 A_2} \right) \nonumber \\&\quad \times F_T(T). \end{aligned}$$

As the constant *T* case reduces to TEGR, $F(T)=T$, with some scaling of physical quantities, we will ignore this case. However, we will state the following result for Kantowski–Sachs geometries admitting a timelike affine frame symmetry.

#### Proposition 5.1

Any teleparallel Kantowski–Sachs geometry, which admits the affine frame symmetry, $\textbf{X}_5 = -\frac{1}{C_0} \partial _t + r \partial _r$ with the additional Lie bracket relations

$$\begin{aligned}{}[\textbf{X}_I, \textbf{X}_5] = 0,\quad [\textbf{X}_4, \textbf{X}_5] = \textbf{X}_4, \end{aligned}$$

is given by the following:

$$\begin{aligned} \psi = 0, \chi = \frac{\pi }{2}, A_3 = \frac{1}{C_0},~ A_2 = e^{C_0 t}, \end{aligned}$$

and is a solution of the constant torsion scalar eqn. with $\rho = p = 0$.

### Non-constant torsion scalar

The torsion scalar is

110 $$\begin{aligned} T = 2\left( \frac{A_{3}'}{A_3} \right) ^2 + \frac{4 A_{3}' A_{2}'}{A_3 A_2} - \frac{2}{A_3^2}. \end{aligned}$$

Writing $F_T(T)$ as $F'(T)$ in this subsection, the algebraically independent components of the symmetric part of the FEs are then:

111a $$\begin{aligned}&\kappa \rho + \frac{F(T)}{2} = \left( T+\frac{2}{A_3^2} \right) F'(T), \end{aligned}$$

111b $$\begin{aligned}&-\kappa ( \rho + P) = \left( \frac{ A_{3}'}{A_3}\right) T'\, F''(T) + \left( \frac{A_{3}''}{A_3} - \frac{A_{3}' A_{2}'}{A_3 A_2} \right) F'(T), \end{aligned}$$

111c $$\begin{aligned}&\left( \left( \ln (A_2)\right) ' - \left( \ln (A_3)\right) ' \right) T'\,F''(T) \nonumber \\&\quad =- \left( \frac{A_{3}' A_{2}'}{A_2 A_3} - \left( \frac{A_{3}'}{A_3} \right) ^2 - \frac{A_{3}''}{A_3} + \frac{A_{2}''}{A_2} - \frac{1}{A_3^2} \right) F'(T). \end{aligned}$$

Comparing with previous discussions of the Kantowski–Sachs *F*(*T*) FEs in the literature [48, 49], we point out that the governing eqns. differ significantly. In particular, the anti-symmetric part of the FEs do not require $F(T) =T$ and the symmetric eqns. have subtle but important differences, such as the inclusion of $2 A_3^{-2}$ in the coefficient of $F'(T)$ in Eq. (111a).

To avoid $F'(T) = 0$ or $F''(T)=0$, the coefficients in the Eq. (111c) cannot vanish (otherwise it would imply either $A_2$ or $A_3$ vanishes, which is not permitted). We may partially integrate $F'(T)$ from this eqn.:

112 $$\begin{aligned} F'(T)= & {} C_0 \exp \left[ \int \frac{\left( -\frac{A_{3}' A_{2}'}{A_2 A_3} + \left( \frac{A_{3}'}{A_3}\right) ^2 + \frac{A_{3}''}{A_3} - \frac{A_{2}''}{A_2} + \frac{1}{A_3^2} \right) }{\left( \ln (A_2)\right) ' - \left( \ln (A_3)\right) '} dt \right] \nonumber \\= & {} C_0 \exp ( B(t)). \end{aligned}$$

Substituting this into Eqs. (111a) and (111b) the FEs become:

113a $$\begin{aligned} \kappa \rho + \frac{F(T)}{2}&= \left( T + \frac{2}{A_3^2} \right) F'(T), \end{aligned}$$

113b $$\begin{aligned} -\kappa (\rho + P)&= \left( \frac{A_3'}{A_3} B' + \frac{A_3''}{A_3} - \frac{A_3' A_2'}{A_3 A_2} \right) F'(T). \end{aligned}$$

#### The vacuum *F*(*T*) equations

In the case of vacuum, where $\rho = P = 0$, we find the following conditions:

114a $$\begin{aligned} \frac{F(T)}{2}&= \left( T + \frac{2}{A_3^2}\right) F'(T), \end{aligned}$$

114b $$\begin{aligned} 0&= \left( \frac{A_3'}{A_3} B' + \left( \frac{A_3'}{A_3} \right) ' + \left( \frac{A_3'}{A_3} \right) ^2 - \frac{A_3' A_2'}{A_3 A_2} \right) F'(T) . \end{aligned}$$

To avoid $F'(T) = 0$, the Eq. (114b) must vanish. The general solution to the Eqs. (114a) and (114b) is possible. However special solutions will be sufficient for our purposes. One solution to this eqn. follows from requiring $A_2=A_3^n$, where $n \in \mathbb {R}$. If $A_3$ is not constant, then the resulting functions and eqns. are:

115a $$\begin{aligned} T =&2(2n+1)\,\left[ \left( \ln \,(A_3)\right) '\right] ^2-2\,A_3^{-2}, \end{aligned}$$

115b $$\begin{aligned} B'=&\frac{\left( 1-n^2\right) \left[ \left( \ln \,(A_3)\right) '\right] ^2-(n-1)\, \frac{A_3''}{A_3}+A_3^{-2}}{(n-1)\,\left( \ln \,(A_3)\right) '}, \end{aligned}$$

115c $$\begin{aligned} 0 =&\left( \ln \,(A_3)\right) '\,B'+\frac{A_3''}{A_3}-n\,\left[ \left( \ln \,(A_3)\right) '\right] ^2, \end{aligned}$$

115d $$\begin{aligned} \frac{F(T)}{2} =&\left( T+\frac{2}{A_3^2}\right) \,F'(T). \end{aligned}$$

Eliminating $B'$, we find the following eqn. for $A_3$:

116 $$\begin{aligned} 0=(2n+1)(n-1)\left( \frac{A_3'}{A_3}\right) ^2-\frac{1}{A_3^2}, \end{aligned}$$

with the corresponding solution:

117 $$\begin{aligned} A_3 = \pm \frac{t}{\sqrt{(2n+1)(n-1)}},\quad n>1. \end{aligned}$$

Finally, writing *T* in terms of these functions, we obtain:

118 $$\begin{aligned} T = \frac{2(2-n)(2n+1)}{t^2}. \end{aligned}$$

In the case where the constant of integration is zero, we find the following eqn. for *F*(*T*):

119 $$\begin{aligned} \frac{F(T)}{2}=&\left( \frac{T}{2-n}\right) F'(T), \end{aligned}$$

which integrates to

120 $$\begin{aligned} F(T) =F_1\,T^{1-\frac{n}{2}}. \end{aligned}$$

## Special similarity case

We wish to study the special case of spherical symmetry with the 3 affine spherical symmetry vectors and the additional fourth affine vector *X* (or $X_4$) (38) which commutes with $\{X_x, X_y, X_z\}$.

To compute the conditions on the frame and the connection, we suppose that the affine vectors, $\{X_I\}_{I=1}^4 = \{ X_x, X_y, X_z, X_4\}$, act on the frame in the following way:

$$\begin{aligned} \mathcal {L}_{\mathbf{X_I}} {\textbf{h}}^a = f_I^{~\hat{i}} \lambda _{\hat{i}~b}^a {\textbf{h}}^b, \end{aligned}$$

where $\lambda _{\hat{i}}$ is defined as before in Eq. (15) and $f_{I}^{~\hat{i}}$ are functions of $re^{-C_0 t}, \theta$ and $\phi$ (where $C_0 \equiv C_3 C_4$; and $C_0\equiv -H_0$ to compare with the TdS solution).

In order to determine $f_I^{\hat{i}}$, we must expand the Lie algebra constants $C^I_{~JK}$ and then solve the resulting DEs from (5a). Using the transformation properties of $\overline{Iso}$ a frame can be chosen where

121 $$\begin{aligned} f_I^{\hat{i}} = \left[ \begin{array}{ccc} \frac{\cos \phi }{\sin \theta } &{} 0 &{} 0 \\ \frac{\sin \phi }{\sin \theta } &{} 0 &{} 0 \\ 0 &{} 0 &{} 0 \\ 0 &{} 0 &{} 0 \\ \end{array} \right] . \end{aligned}$$

Thus, to determine the conditions of the inclusion of the new symmetry generator, we need only examine the conditions

122 $$\begin{aligned} \mathcal {L}_{\textbf{X}_4} {\textbf{h}}^a = 0. \end{aligned}$$

Solving the resulting eqns., we find that

123 $$\begin{aligned}{} & {} A_1 (t,r) = f_1(z),\quad A_2(t,r) = \frac{f_2(z)}{r}, \nonumber \\{} & {} A_3(t,r) = f_3(z), \end{aligned}$$

where $z \equiv r e^{-C_0 t}$. Due to the simple form of $f_I^{~\hat{i}}$, the Lie derivative of the connection gives a simple condition, namely that

124 $$\begin{aligned} \mathcal {L}_{\mathbf{X_4}} \omega ^a_{~bc} = \mathbf{X_4 } ( \omega ^a_{~bc}) = 0. \end{aligned}$$

Therefore, the arbitrary functions in the spin-connection’s components must be functions of the form:

125 $$\begin{aligned} \chi (t,r) = \chi (z), \quad \psi (t,r) = \psi (z). \end{aligned}$$

We now have a resulting diagonal metric *g* by substituting the coframe components, where $\mathcal {L}_{\textbf{X}}\,g = 0$ as required.

### Similarity

We can modify the frame ansatz on the vierbein functions:

126 $$\begin{aligned} A_1 = r^\lambda f_1(z), \quad A_2 = r^{\lambda -1} f_2(z), \quad A_3 = r^\lambda f_3(z), \end{aligned}$$

where if $\lambda \ne 0$, $\textbf{X} = \frac{1}{C_0} \partial _t + r \partial _r$ corresponds to a homothetic vector with $\mathcal {L}_{\textbf{X}}\,g = 2\lambda \,g$, while if $\lambda = 0$ this is an affine frame symmetry and hence a Killing vector.

$\mathcal {L}_{\textbf{X}}\,\omega$ can be calculated by considering the components for $\omega _{ab}$ defined as follows:

127 $$\begin{aligned} \omega _{12}=&-W_5\,dt-W_6\,dr , \omega _{13} = -W_7 d\theta -W_8\,\sin \theta \,d\phi , \nonumber \\ \omega _{14} =&W_8\,d\theta -W_7\,\sin \theta \,d\phi ,\nonumber \\ \omega _{23}=&W_3\,d\theta + W_4\,\sin \theta d\phi , \omega _{24} = -W_4\,d\theta +W_3\,\sin \theta \,d\phi ,\nonumber \\ \omega _{34} =&W_1\,dt+W_2\,dr-\cos \theta \,d\phi , \end{aligned}$$

from which we find that $\mathcal {L}_{\textbf{X}}\,\omega =0$ for all values of $\lambda$ [16]. For $\lambda =0$, we still get $\mathcal {L}_{\textbf{X}}\,g=0$ and $\mathcal {L}_{\textbf{X}}\,\omega ^a_{~bc}=0$ as expected.

Note that

128 $$\begin{aligned} r\,\partial _r\,T= & {} r^{-2\lambda }\,\left( -2\,\lambda \,T_0(z)+z\,\partial _z\,T_0(z)\right) ,\nonumber \\ z\,\partial _z\,T= & {} r^{-2\lambda }\,z\,\partial _z\,T_0(z). \end{aligned}$$

For the general situation ($\lambda \ne 0$), we have that the torsion scalar $T=T(z,r)$ will also contain terms in $r^{\lambda }$; $T(z,r)=\frac{T_0(z)}{r^{2\lambda }}$.

The general torsion scalar (consistent with the form of these expressions) is given explicitly by:

129 $$\begin{aligned}&T(z, r)\equiv \frac{T_0(z)}{r^{2\lambda }} = \frac{1}{r^{2\lambda }f_1^2\,f_2^2\,f_3^2}\nonumber \\&\quad \times \Bigg [-8\,f_1\,f_2\,\cos \chi \,\Bigg (\Bigg [f_1\, \left( \lambda \,f_3+\frac{z\,f_3'}{2}\right) \nonumber \\&\quad +\frac{z\,f_3\left( \psi '\,C_0\,f_2+f_1'\right) }{2}\Bigg ]\cosh \psi \nonumber \\&\quad +\frac{z\,\sinh \psi }{2}\left[ f_1\,f_3\,\psi '+C_0\,\left( f_2\,f_3\right) '\right] \Bigg )\nonumber \\&\quad +4\,z\,\sin \chi \,\cosh \psi \,\chi '\,f_1^2\,f_2\,f_3\nonumber \\&\quad +4\,z\,\sinh \psi \,\sin \chi \,\chi '\,C_0\,f_1\,f_2^2\,f_3\nonumber \\&\quad +f_1^2\,\left[ -6\lambda ^2\,f_3^2-8\lambda \,z\,f_3\,f_3'-2\,z^2\,f_3'^2-2\,f_2^2\right] \nonumber \\&\quad -4\, f_1\,f_3\left[ \lambda \,C_0\,z\,f_2\,f_3\,\psi '+f_1'\left( \lambda \,z\,f_3+z^2\,f_3'\right) \right] \nonumber \\&\quad +2\,C_0^2\,z^2\,f_2\,f_3'\left( f_2\,f_3'+2\,f_3\,f_2'\right) \Bigg ] . \end{aligned}$$

### The antisymmetric FEs

Therefore, the non-trivial antisymmetric FEs will be as follows ($\lambda \ne 0$):

130a $$\begin{aligned} 0&= z\,\left( \ln \,T_0(z)\right) '\left[ \cos \chi \,\left( C_0\,f_2\,\cosh \psi +f_1\,\sinh \psi \right) \right. \nonumber \\&\quad \left. +\lambda \,C_0\,f_3\right] -2\,\lambda \,\left[ \cos \chi \,\left( f_1\,\sinh \psi \right) -C_0\,z\,f_3'\right] , \end{aligned}$$

130b $$\begin{aligned} 0&=\sin \chi \,\Bigg [z\,\left( \ln \,T_0(z)\right) '\left[ C_0\,f_2\,\sinh \psi \right. \nonumber \\&\quad \left. +f_1\,\cosh \psi \right] -2\,\lambda \, \left[ f_1\,\cosh \psi \right] \Bigg ] , \end{aligned}$$

where $\chi =\chi (z)$, $\psi =\psi (z)$ and the $f_i=f_i(z)$ are defined earlier.

There are two subsolutions of Eqs. (130a) and (130b) for $T_0(z)\ne 0$ as follows:

1. $\sin \,\chi (z)=0$: $\cos \,\chi (z)=\delta =\pm 1$, $\chi = k\pi$ and the Eq. (130a) is:
  131 $$\begin{aligned} \left( \ln \,T_0(z)\right) '=2\,\lambda \,\frac{\left[ \delta \,\left( f_1\,\sinh \psi \right) -C_0\,z\,f_3'\right] }{z\,\left[ \delta \, \left( C_0\,f_2\,\cosh \psi +f_1\,\sinh \psi \right) +\lambda \,C_0\,f_3\right] }. \end{aligned}$$
  The general solution of Eq. (131) can be obtained by integration:
  132 $$\begin{aligned} T_0(z)&=T_0(0)\,\exp \nonumber \\&\quad \left[ \!2 \lambda \!\int _0^{z}\! dz\!\frac{\left[ \delta \,\left( f_1\,\sinh \psi \right) -C_0\,z\,f_3'\right] }{z\,\left[ \delta \,\left( C_0\,f_2\,\cosh \psi +f_1\,\sinh \psi \right) +\lambda \,C_0\,f_3\right] }\!\right] . \nonumber \\ \end{aligned}$$
  The four symmetric FEs will be used to determine the functions $\psi (z)$ and the $f_i(z)$ where $i=1,\,2,\,3$.
2. If $\sin \,\chi (z)\ne 0$, we get that:
  133a $$\begin{aligned}&z\,\left( \ln \,T_0(z)\right) '\,\left[ \cos \chi \,\left( C_0\,f_2\,\cosh \psi +f_1\,\sinh \psi \right) +\lambda \,C_0\,f_3\right] \nonumber \\&\quad =2\,\lambda \,\left[ \cos \chi \,\left( f_1\,\sinh \psi \right) -C_0\,z\,f_3'\right] , \end{aligned}$$
  133b $$\begin{aligned}&z\,\left( \ln \,T_0(z)\right) '\,\left[ C_0\,f_2\,\sinh \psi +f_1\,\cosh \psi \right] =2\, \lambda \,\left[ f_1\,\cosh \psi \right] . \end{aligned}$$
  We substitute Eq. (133a) into Eq. (133b) and we obtain the relation:
  134 $$\begin{aligned} \cos \chi =-\left[ \frac{\sinh \psi }{f_1}\left( C_0\,z\,f_3'\right) +\frac{\cosh \psi }{f_2}\, \left( z\,f_3'+\lambda \,f_3\,\right) \right] . \end{aligned}$$
  Here again $z=z(t,r)$ is still a function of *t* and *r*. It remains to substitute Eq. (134) into the symmetric FEs as well as into Eq. (132) to obtain the specific form of $T_0(z)$ applicable for this 2nd subsolution.

### The symmetric FEs

The general symmetric FEs are:

135a $$\begin{aligned} 0= & {} 2\,r^{-4\lambda }\,F''(T)\,\left[ h_1\,T_0'(z)+m_1\,T_0(z)\right] \nonumber \\{} & {} + g_1\,r^{-2\lambda }\,F'(T), \end{aligned}$$

135b $$\begin{aligned} \kappa \,P -\frac{F(T)}{2}= & {} 2\,r^{-4\lambda }\,F''(T)\,h_2\,T_0'(z)+2\,g_2\,r^{-2\lambda }\,F'(T), \nonumber \\ \end{aligned}$$

135c $$\begin{aligned} \kappa \,\rho + \frac{F(T)}{2}= & {} 4\,r^{-4\lambda }\,F''(T)\,\left[ h_3\,T_0'(z)+m_3\,T_0(z)\right] \nonumber \\{} & {} +2\,g_3\,r^{-2\lambda }\,F'(T), \end{aligned}$$

135d $$\begin{aligned} 0= & {} r^{-4\lambda }\,F''(T)\,\left[ h_4\,T_0'(z)+m_4\,T_0(z)\right] \nonumber \\{} & {} + g_4\,r^{-2\lambda }\,F'(T), \end{aligned}$$

where the explicit forms of the functions $h_1,\,m_1,$ etc... in the symmetric FE above in the case of the two subsolutions are given explicitly in Appendix B.

By taking the Eqs. (135a) and (135d), we obtain the following expression:

136 $$\begin{aligned} -\frac{F''(T)\,r^{-2\lambda }}{F'(T)}&=\frac{g_1}{2\left[ h_1\,T_0'(z)+m_1\,T_0(z)\right] } \nonumber \\&=\frac{g_4}{\left[ h_4\,T_0'(z)+m_4\,T_0(z)\right] }, \end{aligned}$$

and hence formally:

137 $$\begin{aligned} T_0(z)=T_0(0)\,\exp \left[ \int _{0}^{z}\,dz\,\frac{2\,g_4\,m_1-g_1\,m_4}{g_1\,h_4-2\,g_4\,h_1}\right] , \end{aligned}$$

which yields the function $T_0(z)$ as a function of $\psi (z)$, $\chi (z)$ and $f_i(z)$. From the FEs expressed by Eqs. (135a) to (135d), for $\lambda \ne 0$, there are restrictions on the form of the function *F*(*T*). Equations (135b) and (135c) become:

138a $$\begin{aligned} \kappa \,P= & {} \left[ \frac{2\,\alpha (z)}{\beta (z)}\,h_2\,T_0'(z) +\left( 2\,g_2+\frac{\alpha (z)}{2}\right) \right] \,r^{-2\lambda }\,F'(T), \nonumber \\ \end{aligned}$$

138b $$\begin{aligned} \kappa \,\rho= & {} \left[ \frac{4\,\alpha (z)}{\beta (z)}\,\left( h_3\,T_0'(z)+m_3\,T_0(z)\right) +\left( 2\,g_3-\frac{\alpha (z)}{2}\right) \right] \nonumber \\{} & {} \times r^{-2\lambda }\,F'(T), \end{aligned}$$

where $\frac{\alpha (z)}{\beta (z)}=\frac{T\,F''(T)}{T_{0}(z)\,F'(T)}$ and the functions $\alpha (z)$ and $\beta (z)$ are defined by Eqs. (135b) and (135c).

Hence the Eqs. (135b) and (135c) are replaced by Eqs. (138a) and (138b) giving *P* and $\rho$ with *F*(*T*). A possible solution of the FEs is that *F*(*T*) is the sum of terms of the form:

139 $$\begin{aligned} F(T)=\sum \, F_{\gamma }(T)= \sum _{\gamma \ge 0}\, F_{0\;\gamma }\,T^{\gamma }, \end{aligned}$$

with possible constraints on $\alpha (z)$ and $\beta (z)$ leading to additional ODEs. *P* and $\rho$ are determined by Eqs. (138a) and (138b).

By adding the Eqs. (138a) and (138b) together, we obtain the following eqn. in term of $r^{-2\lambda }\,F'(T)$:

140 $$\begin{aligned} \kappa \,\left( P + \rho \right)= & {} 2\,\left[ -g_4\,\left[ \frac{\left( h_2 +2\,h_3\right) \,T_0'(z) + \left( 2\,m_3\right) \,T_0(z)}{h_4\,T_0'(z)+m_4\,T_0(z)}\right] \right. \nonumber \\{} & {} \left. +\left( g_2+g_3\right) \right] \,r^{-2\lambda }\,F'(T),\nonumber \\\equiv & {} 2\,H(z)\,r^{-2\lambda }\,F'(T). \end{aligned}$$

We will study the different *H*(*z*) giving the Eq. (140) for the general symmetric FEs as well as for the two subsolutions.

For the situation without utilizing the antisymmetric FE solutions, we obtain:

141 $$\begin{aligned} H(z) =&-2\,C_0\,z^2\,\Bigg [\left( \ln \,f_3\right) '\,\left[ \ln \left( \frac{f_1\,f_2}{z\,f_3'}\right) \right] '+\left[ \ln \left( z^{\lambda }\right) \right] '\,\left[ \ln \, f_2\right] '\Bigg ]\nonumber \\&\times \Bigg [2\lambda \,T_0(z) \left[ \frac{z\,f_3'+\lambda \,f_3}{f_2^2}+\frac{1}{f_2}\,\cosh \psi \,\cos \chi \right] \nonumber \\&+\left[ \left( \frac{C_0\,\sinh \psi }{f_1}-\frac{\cosh \psi }{f_2}\right) \,\cos \chi \right. \nonumber \\&\left. -\left( \frac{C_0^2}{f_1^2} +\frac{1}{f_2^2}\right) \,z\,f_3'-\frac{\lambda \,f_3}{f_2^2}\right] \,z\,T_0'(z)\Bigg ]\nonumber \\&\times \,\Bigg [-z\,T_0'(z)\,\left[ \left( C_0\,f_2\,\cosh \psi -f_1\,\sinh \psi \right) \,\cos \chi \right. \nonumber \\&\left. +\lambda \,C_0\,f_3+2\,C_0\,z\,f_3'\right] \nonumber \\&+\lambda \,T_0(z)\,\left[ 2\,C_0\,z\,f_3'-f_1\, \sinh \psi \,\cos \chi \right] \Bigg ]^{-1}\nonumber \\&+\Bigg [z^2\,\left( \ln \,f_3\right) '\,\left( \frac{C_0^2}{f_1^2}+\frac{1}{f_2^2}\right) \left[ \ln \left( \frac{f_1\,f_2}{z\,f_3'}\right) \right] '\nonumber \\&+\frac{\lambda \,z}{f_2^2}\,\left[ \ln \left( f_1\,f_2\,z^{\lambda }\right) \right] '\Bigg ] \end{aligned}$$

When the two subsolutions of the antisymmetric FE are utilized these expressions become much simpler. In particular, Eq. (141) gives $H(z) \ne 0$ in general (for $\lambda \ne 0$). We note that when $\lambda = 0$ (special isometry case), we find that $H(z) = 0$, so that $\rho + P = 0$, so that from conservation laws $\rho = \Lambda _0 =$ constant and $P = - \Lambda _0$ [50].

For the $H(z) \ne 0$ case, one must take into account the conservation laws which can be summarized by the following relation:

142 $$\begin{aligned} \left( \rho +P\right)= & {} -\frac{f_1}{\left( \lambda \,f_1+z\,f_1'\right) }\,\left( r\,\partial _r\,P(t,r)\right) \nonumber \\= & {} \frac{f_2\,f_3}{C_0\,z\,\left( 2\,f_2\,f_3'+f_2'\,f_3\right) }\,\left( \partial _t\,\rho (t,r)\right) . \end{aligned}$$

We obtain from Eq. (142) a relation of the form:

143 $$\begin{aligned} \left( r\,\partial _r\,P(t,r)\right) \equiv -\Sigma (z)\,\left( \partial _t\,\rho (t,r)\right) . \end{aligned}$$

We must solve Eq. (143) for $\Sigma (z)$ to determine the kind of fluid corresponding to the *H*(*z*) obtained. To make further progress, we could assume an equation of state of the form $P=P(\rho )$ (or more generally for $\rho \equiv \sum _{n= 0}\,\rho _i(z)\,r^{-2n\,\lambda }$ and $P\equiv \sum _{n= 0}\,P_i(z)\,r^{-2n\,\lambda }$, eqns. of state for each component $\rho _i$, $P_i$) leading to further ODEs. Alternatively, we could investigate a particular *F*(*T*).

### A particular quadratic *F*(*T*) theory

As an example, let us work with the following quadratic function of *T* [51]:

144 $$\begin{aligned} F(T) = -\Lambda +T+\gamma \,T^2, \end{aligned}$$

where $\gamma$ is a constant (we could also consider a cubic function or an exponential function $F(T) = \frac{1}{2\gamma }\,\left[ \exp \left( 2\gamma \,T\right) -1\right]$). The symmetric FEs for the 1st subsolution is

145a $$\begin{aligned} 0 =&4\,r^{-4\lambda }\,\gamma \,\left[ h_1\,T_0'(z)+m_1\,T_0(z)\right] + g_1\,r^{-2\lambda }\, \Bigg (1+2\gamma \,T_0(z)\,r^{-2\lambda }\Bigg ), \end{aligned}$$

145b $$\begin{aligned} \kappa \,P =&\frac{1}{2}\Bigg (-\Lambda +T_0(z)\,r^{-2\lambda }+\gamma \,T_0^2(z)\,r^{-4\lambda }\Bigg )\nonumber \\&\quad +4\,r^{-4\lambda }\,\gamma \,h_2\,T_0'(z)+2\,g_2\,r^{-2\lambda }\,\Bigg (1+2\gamma \,T_0(z)\,r^{-2\lambda }\Bigg ), \end{aligned}$$

145c $$\begin{aligned} \kappa \,\rho =&-\frac{1}{2}\Bigg (-\Lambda +T_0(z)\,r^{-2\lambda }+\gamma \,T_0^2(z)\,r^{-4\lambda }\Bigg )\nonumber \\&\quad +8\,r^{-4\lambda }\,\gamma \,\left[ h_3\,T_0'(z)+m_3\,T_0(z)\right] +2\,g_3\,r^{-2\lambda }\nonumber \\&\quad \times \Bigg (1+2\gamma \,T_0(z)\,r^{-2\lambda }\Bigg ), \end{aligned}$$

145d $$\begin{aligned} 0 =&2\,r^{-4\lambda }\,\gamma \,\left[ h_4\,T_0'(z)+m_4\,T_0(z)\right] \nonumber \\&\quad + g_4\,r^{-2\lambda }\, \Bigg (1+2\gamma \,T_0(z)\,r^{-2\lambda }\Bigg ) . \end{aligned}$$

The Eqs. (145a) and (145d) give the following relation:

146 $$\begin{aligned} \frac{r^{2\lambda }}{2\gamma }\Bigg (1+2\gamma \,T_0(z)\,r^{-2\lambda }\Bigg )&=\frac{2\left[ h_1\,T_0'(z)+m_1\,T_0(z)\right] }{g_1}\nonumber \\&=\frac{\left[ h_4\,T_0'(z)+m_4\,T_0(z)\right] }{g_4}. \end{aligned}$$

With the Eq. (146), the Eqs. (145b) and (145c) become explicitly:

147a $$\begin{aligned} \kappa \,P&= \frac{1}{2}\Bigg (-\Lambda +T_0(z)\,r^{-2\lambda }\Bigg )+4\,\gamma \,r^{-4\lambda }\, \nonumber \\&\quad \times \Bigg [\left( h_2+\frac{g_2}{g_4}\,h_4\right) \,T_0'(z)+\left( \frac{g_2}{g_4}\,m_4\right) \,T_0(z) +\frac{T_0^2(z)}{8}\Bigg ], \nonumber \\ \end{aligned}$$

147b $$\begin{aligned} \kappa \,\rho&= -\frac{1}{2}\Bigg (-\Lambda +T_0(z)\,r^{-2\lambda }\Bigg )+4\,\gamma \,r^{-4\lambda }\, \Bigg [\left( 2 h_3+\frac{g_3}{g_4}\,h_4\right) \nonumber \\&\quad \times T_0'(z) +\left( 2 m_3+\frac{g_3}{g_4}\,m_4\right) \,T_0(z)-\frac{T_0^2(z)}{8}\Bigg ]. \end{aligned}$$

By adding the Eqs. (147a) and (147b) into Eq. (141), we obtain the following eqn.:

148 $$\begin{aligned} \kappa \,\left( P + \rho \right)&= 4\,\gamma \,r^{-4\lambda }\,\Bigg [\left( \frac{\left( g_2+g_3\right) }{g_4}\, h_4+h_2+2 h_3\right) \,T_0'(z)\nonumber \\&\quad +\left( \frac{\left( g_2+g_3\right) }{g_4}\,m_4+2 m_3\right) \,T_0(z)\Bigg ],\nonumber \\&= 4\,\gamma \,r^{-4\lambda }\,\tilde{H}(z), \end{aligned}$$

where $\tilde{H}(z)$ is the original *H*(*z*) of Eq. (140) multiplied by some terms inside $F'(T)$ (derivative of Eq. (144) terms). The Eq. (148) describes the eqns. (138a) and (138b) solution for the quadratic theory described by Eq. (144).

### Simple power-law solutions

By using the power-law ansatz $f_1=a_0\,z^a$, $f_2=b_0\,z^b$, $f_3=c_0\,z^c$ and $\psi =e_0\,z^e$ with $P=P(z,r)$ and $\rho =\rho (z,r)$, the conservation laws become:

149a $$\begin{aligned}{} & {} -\left( \lambda +a\right) \left( \rho (z,r)+P(z,r)\right) = \left( z\,\partial _z\,P(z,r)+r\,\partial _r\,P(z,r)\right) , \nonumber \\ \end{aligned}$$

149b $$\begin{aligned}{} & {} \left( 2c+b\right) \left( z\,\partial _z\,P(z,r)+r\,\partial _r\,P(z,r)\right) = \left( \lambda +a\right) \,z\,\partial _z\,\rho (z,r), \nonumber \\ \end{aligned}$$

where $\partial _r P(t,r)=\frac{z}{r}\,\partial _z\,P(z,r)+\partial _r\,P(z,r)$ and $\partial _t \rho (t,r)=-C_0\,z \partial _z\,\rho (z,r)$. The following cases arise:

1. $b=-2c$: Leading to solutions with a static fluid $P=P(r)$ and $\rho =\rho (r)$.
2. $a= -\lambda$: We obtain that $P=P(t)$ and there is no constraint on $\rho$.

Additional solutions are possible with further assumptions on the form of *F*(*T*) or the perfect fluid. For example, assuming a perfect fluid $P(z,r)=\alpha \,\rho (z,r)$ with a linear eqn. of state, we obtain:

150a $$\begin{aligned}{} & {} -\left( \lambda +a\right) \left( 1+\alpha \right) \rho (z,r) = \alpha \left( z\,\partial _z\, \rho (z,r)+r\,\partial _r\,\rho (z,r)\right) , \nonumber \\ \end{aligned}$$

150b $$\begin{aligned}{} & {} \alpha \left( 2c+b\right) \left( z\,\partial _z\,\rho (z,r)+r\,\partial _r\,\rho (z,r)\right) = \left( \lambda +a\right) \,z\,\partial _z\,\rho (z,r), \nonumber \\ \end{aligned}$$

where $-1 < \alpha \le 1$. We find the solution is:

151 $$\begin{aligned} \rho (z,r)= & {} \rho (0,r)\,z^{-\left( 2c+b\right) \,\left( 1+\alpha \right) }, \end{aligned}$$

where $\rho (0,r)=g(r)$ and we find that:

152 $$\begin{aligned} \rho (0,r)=g(r)=g(0)\,r^{\frac{\left( 1+\alpha \right) }{\alpha }\left[ \alpha \left( 2c+b\right) -\left( \lambda +a\right) \right] }. \end{aligned}$$

Further conditions can then be obtained from the FEs after solving the conservation laws. Typical solutions lead to power-law expressions for *F*(*T*) of the form $F(T) =-\Lambda +\gamma \,T^{\beta }$.

We note that all teleparallel solutions in this section are new.

## Discussion and conclusions

In teleparallel *F*(*T*) gravity, the geometrical quantities of interest (is the torsion computed from) the frame (or co-frame ${\textbf{h}}^a$) and a zero curvature, metric compatible, spin connection ${{{\varvec{\omega }}}}^a_{~b}$. When *F*(*T*) teleparallel gravity is defined in a gauge invariant manner, the ensuing field equations are fully Lorentz covariant. Here we are interested in teleparallel spherically symmetric solutions to the *F*(*T*) teleparallel gravity FEs which are of particular physical importance.

To construct such gravitational models in teleparallel gravity, we first needed to determine the general forms for the the frame (or co-frame) and the spin connection that respect the assumed spherically symmetric (affine) symmetries. Since there is a non-trivial linear isotropy group, the determination of the group of affine symmetries necessarily requires the solution to a system of inhomogeneous DEs (3). Following the approach outlined in [16], which relies on the existence and determination of a class of invariantly defined frames, it then follows from Eqs. (3a) and (3b) that the Lie derivative of the torsion tensor and its covariant derivatives with respect to the affine frame symmetries $\mathbf{X_i}$ are identically zero.

We have presented the (diagonal) frame (or co-frame ${\textbf{h}}^a$) (19) and the metric compatible spin connection (${{{\varvec{\omega }}}}^a_{~b})$ (20) pair that describes the most general spherically symmetric teleparallel geometry, and that also satisfies the flatness condition (21). This is a general geometric result and applies to all theories formulated with respect to a teleparallel geometry, and not just *F*(*T*) teleparallel gravity. As an application, we then employ this general frame/spin connection pair to study the *F*(*T*) spherically symmetric teleparallel gravity field equations. In the general case we presented the solutions of the antisymmetric FEs (which split into two cases, which we consequently considered separately), and derived and subsequently analysed the resulting symmetric FEs.

In order to further study the applications of spherically symmetric teleparallel models in *F*(*T*) teleparallel gravity, we studied 3 subcases in which there is an additional affine symmetry (i.e., models with a 4-dimensional Lie group of affine symmetries) so that the FEs reduce to a system of ordinary differential equations (ODEs) suitable for further investigation.

First we studied static spherically symmetric spacetimes with an additional affine static spherically symmetric generator. We solved the antisymmetric FEs (48) and derived the full set of symmetric FEs (24a)–(24c) and analysed their properties. In general, we assumed that the source is a perfect fluid and utilized a particular coordinate choice ($A_3=r$), and we also specifically investigated the vacuum FEs and obtained a number of new results. In particular, we studied power law solutions in detail and derived a number of new solutions (which, in some cases, reduce to the known power-law solution of Golovnev–Guzman [22]).

We then considered an additional affine frame symmetry in order to expand the affine frame symmetry group of the spherically symmetric case to that of a spatially homogeneous Kantowski–Sachs geometry. A number of new results were obtained. Finally, we studied the special case of spherical symmetry with the 3 affine spherical symmetry vectors and an additional fourth affine symmetry vector (38) which commutes with them, and its similarity generalization. Again we studied the antisymmetric and symmetric FEs in this new class of teleparallel similarity spacetimes. We focused on simple power law models and obtained some new solutions.

## Data Availability

This manuscript has no associated data or the data will not be deposited. [Authors’ comment: No datasets were generated or analysed during the current study.]

## Appendix A: The field equations

Let us present an alternative form of the FEs for *F*(*T*) teleparallel gravity. The FE can be decomposed into antisymmetric and symmetric parts:

153a $$\begin{aligned} 0= & {} F''(T)S_{[ab]}^{~~~~\nu } \partial _{\nu } T, \end{aligned}$$

153b $$\begin{aligned} \kappa \varTheta _{(ab)}= & {} \frac{1}{2}g_{ab}\left[ F(T)-TF'(T)\right] \nonumber \\{} & {} + F''(T)S_{(ab)}^{~~~~\nu } \partial _{\nu } T+F'(T)G_{ab}, \end{aligned}$$

where $G_{ab}$ is the usual Einstein tensor calculated from the metric and where it has been assumed that $\varTheta _{[ab]}=0$.

In the spherically symmetric case studied here with coframe given by Eq. (19) and spin connection given by Eqs. (20) and (21) the torsion scalar has the form

154 $$\begin{aligned} T&=-4\left( \frac{\partial _t \chi }{A_1}\right) \left( \frac{\sin (\chi )\sinh (\psi )}{A_3}\right) +4\left( \frac{\partial _r \chi }{A_2}\right) \left( \frac{\sin (\chi )\cosh (\psi )}{A_3}\right) \nonumber \\&\quad +4\left( \frac{\partial _t \psi }{A_1}\right) \Bigg (\frac{\cos (\chi )\cosh (\psi )}{A_3}\nonumber \\&\quad +\frac{\partial _r A_3}{A_2A_3}\Bigg )-4\left( \frac{\partial _r \psi }{A_2}\right) \left( \frac{\cos (\chi )\sinh (\psi )}{A_3}+\frac{\partial _t A_3}{A_1A_3}\right) \nonumber \\&\quad -4\left( \frac{\partial _r A_1}{A_2 A_1}+\frac{\partial _r A_3}{A_2 A_3}\right) \left( \frac{\cos (\chi )\cosh (\psi )}{A_3}\right) \nonumber \\&\quad +4\left( \frac{\partial _t A_2}{A_1 A_2}+\frac{\partial _t A_3}{A_1 A_3}\right) \left( \frac{\cos (\chi )\sinh (\psi )}{A_3}\right) -2\left( \frac{\partial _r A_3}{A_2 A_3}\right) ^2\nonumber \\&\quad +2\left( \frac{\partial _t A_3}{A_1 A_3}\right) ^2 -4\left( \frac{\partial _r A_1}{A_2 A_1}\right) \left( \frac{\partial _r A_3}{A_2 A_3}\right) \nonumber \\&\quad +4\left( \frac{\partial _t A_2}{A_1 A_2}\right) \left( \frac{\partial _t A_3}{A_1 A_3}\right) -\frac{2}{A_3^2}. \end{aligned}$$

Assuming a co-moving fluid having energy density $\rho$ and pressure *P*, the antisymmetric part of the FEs yield the following two eqns.

155a $$\begin{aligned} 0= & {} F''(T)\left[ \left( \frac{\partial _r T}{A_2}\right) \left( \frac{\cos (\chi )\,\sinh (\psi )}{A_3} +\frac{\partial _t A_3}{A_1 A_3}\right) \right. \nonumber \\{} & {} \left. -\left( \frac{\partial _t T}{A_1}\right) \left( \frac{\cos (\chi )\,\cosh (\psi )}{A_3} +\frac{\partial _r A_3}{A_2A_3}\right) \right] ,\quad \end{aligned}$$

155b $$\begin{aligned} 0= & {} \frac{F''\left( T\right) \,\sin (\chi )}{A_3}\,\left[ \left( \frac{\partial _r\,T}{A_2}\right) \cosh (\psi ) -\left( \frac{\partial _t\,T}{A_1}\right) \sinh (\psi )\right] .\nonumber \\ \end{aligned}$$

The symmetric part of the FEs can be expressed in the following way

156a $$\begin{aligned}&\kappa \rho =-\frac{1}{2}\left( F(T)-TF'(T)\right) -2F''(T)\left( \frac{\partial _r T}{A_2}\right) \nonumber \\&\quad \times \left( \frac{\cos (\chi )\cosh (\psi )}{A_3}+\frac{\partial _r A_3}{A_2A_3}\right) +F'(T)\biggl [-2\frac{\partial _r^2 A_3}{A_2^2A_3}\nonumber \\&\quad +2\left( \frac{\partial _r A_2}{A_2^2}\right) \left( \frac{\partial _r A_3}{A_2 A_3}\right) -\left( \frac{\partial _r A_3}{A_2A_3}\right) ^2+\frac{1}{A_3^2}\nonumber \\&\quad +2\left( \frac{\partial _t A_2}{A_1 A_2}\right) \left( \frac{\partial _t A_3}{A_1 A_3}\right) +\left( \frac{\partial _t A_3}{A_1 A_3}\right) ^2\biggr ] , \end{aligned}$$

156b $$\begin{aligned}&0=-F''(T)\left[ \left( \frac{\partial _r T}{A_2}\right) \left( \frac{\cos (\chi )\sinh (\psi )}{A_3} +\frac{\partial _t A_3}{A_1A_3}\right) \right. \nonumber \\&\quad \left. +\left( \frac{\partial _t T}{A_2}\right) \left( \frac{\cos (\chi )\cosh (\psi )}{A_3} +\frac{\partial _r A_3}{A_2A_3}\right) \right] \nonumber \\&\quad \ +F'(T)\biggl [-2\frac{\partial _t\partial _r A_3}{A_1A_2A_3} +2\left( \frac{\partial _r A_1}{A_1A_2}\right) \left( \frac{\partial _t A_3}{A_1 A_3}\right) \nonumber \\&\quad +2\left( \frac{\partial _r A_3}{A_2A_3}\right) \left( \frac{\partial _t A_2}{A_1A_2}\right) \biggr ], \end{aligned}$$

156c $$\begin{aligned}&\quad \kappa P=\frac{1}{2}\left( F(T)-TF'(T)\right) -2F''(T)\left( \frac{\partial _t T}{A_1}\right) \left( \frac{\cos (\chi )\sinh (\psi )}{A_3}\right. \nonumber \\&\quad \left. +\frac{\partial _t A_3}{A_1A_3}\right) +F'(T)\biggl [2\left( \frac{\partial _r A_1}{A_2A_1}\right) \left( \frac{\partial _r A_3}{A_2 A_3}\right) +\left( \frac{\partial _r A_3}{A_2A_3}\right) ^2 -\frac{1}{A_3^2}\nonumber \\&\quad +2\left( \frac{\partial _t A_1}{A_2A_1}\right) \left( \frac{\partial _t A_3}{A_1 A_3}\right) -\left( \frac{\partial _t A_3}{A_1A_3}\right) ^2 -2\left( \frac{\partial _t^2 A_3}{A_1^2A_3}\right) \biggr ], \end{aligned}$$

156d $$\begin{aligned}&\frac{\kappa }{2} (\rho +3P)=\frac{1}{2}\left( F(T)-TF'(T)\right) \nonumber \\&\quad +F''(T)\biggl [ \left( \frac{\partial _r T}{A_2}\right) \left( \frac{\partial _r A_1}{A_1A_2}-\frac{\partial _t \psi }{A_1}\right) + \left( \frac{\partial _t T}{A_1}\right) \nonumber \\&\quad \times \Bigg (-2\frac{\cos (\chi )\sinh (\psi )}{A_3}-2\frac{\partial _t A_3}{A_1A_3} -\frac{\partial _t A_2}{A_1A_2}+\frac{\partial _r \psi }{A_2}\Bigg )\biggr ]\nonumber \\&\quad +F'(T)\biggl [\frac{\partial _r^2 A_1}{A_2^2A_1} -\left( \frac{\partial _r A_1}{A_2A_1}\right) \left( \frac{\partial _r A_2}{A_2A_2}\right) \nonumber \\&\quad +2\left( \frac{\partial _r A_1}{A_2A_1}\right) \left( \frac{\partial _r A_3}{A_2 A_3}\right) -\frac{\partial _t^2 A_2}{A_1^2A_2} -2\frac{\partial _t^2 A_3}{A_1^2A_3}\nonumber \\&\quad +\left( \frac{\partial _t A_1}{A_1^2}\right) \left( \frac{\partial _t A_2}{A_1A_2}\right) +2\left( \frac{\partial _t A_1}{A_1^2}\right) \left( \frac{\partial _t A_3}{A_1A_3}\right) \biggr ]. \end{aligned}$$

The eqns. above can be supplemented with the energy-momentum conservation eqns.

157a $$\begin{aligned} 0= & {} (\rho +P)\left( \frac{\partial _t{A_2}}{A_2}+2\frac{\partial _t A_3}{A_3}\right) +\partial _t \rho , \end{aligned}$$

157b $$\begin{aligned} 0= & {} (\rho +P)\left( \frac{\partial _rA_1}{A_1}\right) +\partial _r P. \end{aligned}$$

## Appendix B: Symmetric FE components for the $\lambda \ne 0$ solutions

We present the functions defined in the symmetric FE in the 2 subsolutions for the general $\lambda \ne 0$ case.

### B.1 1st subsolution

For the case where $\sin \chi (z)=0$ and $\cos \chi =\delta$, the Eqs. (135a)–(135d) components are:

158a $$\begin{aligned} g_1&= \frac{1}{f_1^3\,f_2^3\,f_3^2} \left[ -\frac{z^2\,f_1\,f_2\,f_3\,f_3''}{2}\left( C_0^2\,f_2^2+f_1^2\right) +\frac{z^2\,f_1\,f_2\,f_3^2}{2}\right. \nonumber \\&\quad \times \left. \left( C_0^2\,f_2\,f_2''-f_1\,f_1''\right) +\frac{z^2\,f_1\,f_2\,f_3'^2}{2}\left( f_1^2-C_0^2\,f_2^2\right) \right. \nonumber \\&\quad \left. +f_3\,f_3'\,\Bigg (\frac{z^2\,f_2\,f_1'}{2}\left( C_0^2\,f_2^2+f_1^2\right) +f_1\left[ \frac{z^2\,f_2'}{2}\,\left( C_0^2\,f_2^2+f_1^2\right) \right. \right. \nonumber \\&\quad \left. \left. +z\,f_2 \left( \left( \lambda -\frac{1}{2}\right) \,f_1^2-\frac{C_0^2\,f_2^2}{2}\right) \right] \Bigg )\right. \nonumber \\&\quad \left. -\frac{f_3^2\,f_1'}{2}\left[ z^2\,f_2'\,\left( C_0^2\,f_2^2-f_1^2\right) +z\,f_1^2\,f_2\right] \right. \nonumber \\&\quad \left. +f_1\,\left[ z\,f_3^2\,f_2'\left( \frac{C_0^2\,f_2^2}{2}+\lambda \,f_1^2\right) +f_1^2\,f_2\left( \lambda ^2\,f_3^2-\frac{f_2^2}{2}\right) \right] \right] , \end{aligned}$$

158b $$\begin{aligned} g_2&=\frac{1}{f_1^3\,f_2^2\,f_3^2}\Bigg [-C_0^2\,z^2\,f_1\,f_2^2\,f_3\,f_3''+z^2\,f_1\,f_3'^2\, \left( f_1^2-C_0^2\,f_2^2\right) \nonumber \\&\quad +f_3'\,\Bigg (2\,z^2\,f_3\,f_1'\left( \frac{C_0^2\,f_2^2}{2}+f_1^2\right) \nonumber \\&\quad +4\,f_1\,\Bigg [-\frac{C_0^2\,z^2\,f_2\,f_3\,f_2'}{4}+z\,\left[ \frac{\delta \,f_1\,f_2}{4} \left( C_0\,f_2\,\sinh \psi \right. \right. \nonumber \\&\quad \left. \left. +f_1\,\cosh \psi \right) +f_3\,\left( -\frac{C_0\,f_2^2}{4}+\lambda \,f_1^2\right) \right] \Bigg ]\Bigg )\nonumber \\&\quad +3\,f_1^2\,f_3\,\Bigg (\frac{2\,z\,f_1'}{3}\left[ \frac{\delta \,f_2\,\cosh \psi }{2} +\lambda \,f_3\right] \nonumber \\&\quad +\frac{z\,f_2\,\psi '}{3}\left[ \delta \,\left( C_0\,f_2\,\cosh \psi +f_1\,\sinh \psi \right) +\lambda \,C_0\,f_3\right] \nonumber \\&\quad +\frac{\delta \,C_0\,z\,f_2\,f_2'\,\sinh \psi }{3}+\lambda \,f_1\,\left[ \frac{2\,\delta \,f_2\,\cosh \psi }{3} +\lambda \,f_3\right] \Bigg )\Bigg ] , \end{aligned}$$

158c $$\begin{aligned} g_3&= \frac{1}{f_1^2\,f_2^3\,f_3^2}\Bigg [-z^2\,f_1^2\,f_2\,f_3\,f_3''+z^2\,f_2\,f_3'^2\, \left( C_0^2\,f_2^2-f_1^2\right) \nonumber \\&\quad +f_3'\,\Bigg (z^2\,f_3\,f_2'\,\left( 2\,C_0^2\,f_2^2+f_1^2\right) \nonumber \\&\quad -4\,f_1\,f_2\,\Bigg [\frac{z^2\,f_3\,f_1'}{4}+z\,\Bigg (\frac{\delta \,f_2}{4}\,\left( C_0\,f_2\, \sinh \psi \right. \nonumber \\&\quad \left. +f_1\,\cosh \psi \right) +f_1\,f_3\,\left( \lambda +\frac{1}{4}\right) \Bigg )\Bigg ]\Bigg )\nonumber \\&\quad -2\,f_1\,f_3\Bigg (-\frac{z\,f_2'}{2}\left( -\delta \,C_0\,f_2^2\,\sinh \psi +\lambda \,f_1\,f_3\right) \nonumber \\&\quad +f_2\,\Bigg [\frac{z\,f_1'}{2}\left( \delta \,f_2\,\cosh \psi +\lambda \,f_3\right) \nonumber \\&\quad +\frac{z\,f_2\,\psi '}{2}\left( \delta \,\left( C_0\,f_2\,\cosh \psi +f_1\,\sinh \psi \right) \right. \nonumber \\&\quad \left. +\lambda \,C_0\,f_3\right) +\lambda \,f_1\,\left( \delta \,f_2\,\cosh \psi +\lambda \,f_3\right) \Bigg ]\Bigg )\Bigg ] , \end{aligned}$$

158d $$\begin{aligned} g_4&= \frac{C_0}{f_1^2\,f_2^2\,f_3}\Bigg [-z^2\,f_1\,f_2\,f_3''+f_3'\,[z^2\,(f_1\,f_2)'-z\,f_1\,f_2]\nonumber \\&\quad +\lambda \,z\,f_1\,f_2'\,f_3\Bigg ] , \end{aligned}$$

158e $$\begin{aligned} h_1&= \frac{1}{f_1^2\,f_2^2\,f_3} \Bigg [-\frac{z^2\,f_1\,f_3\,f_1'}{4} +\frac{C_0^2\,z^2\,f_2\,f_3\,f_2'}{4}-\frac{z\,f_1}{2}\nonumber \\&\quad \times \left[ \frac{\delta \,f_2}{2}\, \left( -C_0\,f_2\,\sinh \psi +f_1\,\cosh \psi \right) +\lambda \,f_1\,f_3\right] \nonumber \\&\quad -\frac{z^2}{4}\,f_3'\,\left( C_0\,^2\,f_2^2+f_1^2\right) \Bigg ] , \end{aligned}$$

158f $$\begin{aligned} h_2&= \frac{C_0}{f_1^2\,f_3}\,\Bigg [\delta \,z\,f_1\,\sinh \psi -C_0\,z^2\,f_3'\Bigg ], \end{aligned}$$

158g $$\begin{aligned} h_3&= -\frac{z}{2\,f_2^2\,f_3}\Bigg [z\,f_3'+\delta \,f_2\,\cosh \psi +\lambda \,f_3\Bigg ], \end{aligned}$$

158h $$\begin{aligned} h_4&=\frac{1}{f_1\,f_2\,f_3}\Bigg [-C_0\,z^2\,f_3'-\frac{z}{2}\,\Big [\delta \,(C_0\,f_2\, \cosh \psi \nonumber \\&-f_1\,\sinh \psi )+\lambda \,C_0\,f_3\Big ]\Bigg ], \end{aligned}$$

158i $$\begin{aligned} m_1&= \frac{\lambda }{f_1\,f_2^2\,f_3}\Bigg [\frac{z\,f_3\,f_1'+C_0\,z\,f_2\,f_3\,\psi '}{2}\nonumber \\&\quad +f_1\,\left[ \frac{\delta \,f_2\,\cosh \psi }{2}+\lambda \,f_3\right] +\frac{z\,f_3'\,f_1}{2}\Bigg ], \end{aligned}$$

158j $$\begin{aligned} m_2&=0 , \end{aligned}$$

158k $$\begin{aligned} m_3&= \frac{\lambda }{f_2^2\,f_3}\Bigg [z\,f_3'+\delta \,f_2\,\cosh \psi +\lambda \,f_3\Bigg ], \end{aligned}$$

158l $$\begin{aligned} m_4&= \frac{1}{f_1\,f_2\,f_3}\Bigg [C_0\,\lambda \,z\,f_3'-\delta \,\lambda \,f_1\,\sinh \psi \Bigg ] . \end{aligned}$$

The $T_0(z)$ expression is the following:

159 $$\begin{aligned}&T_0(z) =\Bigg [ \frac{1}{f_1^2\,f_2^2\,f_3^2}\Bigg [-8\,f_1\,f_2\,\delta \,\Bigg (\left[ f_1\,\left( \lambda \,f_3 +\frac{z\,f_3'}{2}\right) \right. \nonumber \\&\quad \left. +\frac{z\,f_3\left( \psi '\,C_0\,f_2+f_1'\right) }{2}\right] \cosh \psi \nonumber \\&\quad +\frac{z\,\sinh \psi }{2}\left[ f_1\,f_3\,\psi '+C_0\,\left( f_2\,f_3\right) '\right] \Bigg )\nonumber \\&\quad +f_1^2\,\left[ -6\lambda ^2\,f_3^2-8\lambda \,z\,f_3\,f_3'-2\,z^2\,f_3'^2-2\,f_2^2\right] \nonumber \\&\quad -4\,f_1\,f_3\left[ \lambda \,C_0\,z\,f_2\,f_3\,\psi '+f_1'\left( \lambda \,z\,f_3+z^2\,f_3'\right) \right] \nonumber \\&\quad +2\,C_0^2\,z^2\,f_2\,f_3'\left( f_2\,f_3'+2\,f_3\,f_2'\right) \Bigg ]\Bigg ]. \end{aligned}$$

### B.2 2nd subsolution

For the case described by Eqs. (134), (135a)–(135d) components are:

160a $$\begin{aligned} g_1&= \frac{1}{f_1^3\,f_2^3\,f_3^2} \left[ -\frac{z^2\,f_1\,f_2\,f_3\,f_3''}{2} \left( C_0^2\,f_2^2+f_1^2\right) +\frac{z^2\,f_1\,f_2\,f_3^2}{2}\left( C_0^2\,f_2\,f_2''-f_1\,f_1''\right) +\frac{z^2\,f_1\,f_2\,f_3'^2}{2}\left( f_1^2-C_0^2\,f_2^2\right) +f_3\,f_3'\right. \nonumber \\&\quad \times \left. \Bigg (\frac{z^2\,f_2\,f_1'}{2}\left( C_0^2\,f_2^2+f_1^2\right) +f_1\left[ \frac{z^2\,f_2'}{2}\, \left( C_0^2\,f_2^2+f_1^2\right) +z\,f_2\left( \left( \lambda -\frac{1}{2}\right) \,f_1^2-\frac{C_0^2\,f_2^2}{2}\right) \right] \Bigg )\right. \nonumber \\&\quad \left. -\frac{f_3^2\,f_1'}{2}\left[ z^2\,f_2'\,\left( C_0^2\,f_2^2-f_1^2\right) +z\,f_1^2\,f_2\right] +f_1\,\left[ z\,f_3^2\,f_2'\left( \frac{C_0^2\,f_2^2}{2}+\lambda \,f_1^2\right) +f_1^2\,f_2\left( \lambda ^2\,f_3^2-\frac{f_2^2}{2}\right) \right] \right] , \end{aligned}$$

160b $$\begin{aligned} g_2&= \frac{1}{f_1^3\,f_2^2\,f_3^2}\Bigg [-C_0^2\,z^2\,f_1\,f_2^2\,f_3\,f_3''+z^2\,f_1\,f_3'^2\, \left( f_1^2-C_0^2\,f_2^2\right) +f_3'\,\Bigg (2\,z^2\,f_3\,f_1'\left( \frac{C_0^2\,f_2^2}{2}+f_1^2\right) \nonumber \\&\quad +4\,f_1\,\Bigg [-\frac{C_0^2\,z^2\,f_2\,f_3\,f_2'}{4}+z\,\Bigg [-\left[ \frac{\sinh \psi }{f_1} \left( C_0\,z\,f_3'\right) +\frac{\cosh \psi }{f_2}\,\left( z\,f_3'+\lambda \,f_3\,\right) \right] \frac{f_1\,f_2}{4}\nonumber \\&\quad \times \left( C_0\,f_2\,\sinh \psi +f_1\,\cosh \psi \right) +f_3\,\left( -\frac{C_0\,f_2^2}{4}+\lambda \,f_1^2\right) \Bigg ]\Bigg ]\Bigg ) +3\,f_1^2\,f_3\,\Bigg (\frac{2\,z\,f_1'}{3}\left[ -\left[ \frac{\sinh \psi }{f_1}\left( C_0\,z\,f_3'\right) +\frac{\cosh \psi }{f_2}\,\left( z\,f_3'+\lambda \,f_3\,\right) \right] \right. \nonumber \\&\quad \times \left. \frac{f_2\,\cosh \psi }{2}+\lambda \,f_3\right] +\frac{z\,f_2\,\psi '}{3}\left[ -\left[ \frac{\sinh \psi }{f_1}\left( C_0\,z\,f_3'\right) +\frac{\cosh \psi }{f_2}\,\left( z\,f_3'+\lambda \,f_3\,\right) \right] (C_0\,f_2\,\cosh \psi +f_1\, \sinh \psi )+\lambda \,C_0\,f_3\right] \nonumber \\&\quad -\left[ \frac{\sinh \psi }{f_1}\left( C_0\,z\,f_3'\right) +\frac{\cosh \psi }{f_2}\,\left( z\,f_3'+\lambda \,f_3\,\right) \right] \frac{C_0\,z\,f_2\,f_2'\,\sinh \psi }{3} -\frac{z\,f_2}{3}\sin \nonumber \\&\quad \times \left( \arccos \left( -\left[ \frac{\sinh \psi }{f_1}\left( C_0\,z\,f_3'\right) +\frac{\cosh \psi }{f_2}\,\left( z\,f_3'+\lambda \,f_3\,\right) \right] \right) \right) \nonumber \\&\quad \times \left( \arccos \left( -\left[ \frac{\sinh \psi }{f_1}\left( C_0\,z\,f_3'\right) +\frac{\cosh \psi }{f_2}\,\left( z\,f_3'+\lambda \,f_3\,\right) \right] \right) \right) ' \left( C_0\,f_2\,\sinh \psi +f_1\,\cosh \psi \right) +\lambda \,f_1\,\left[ -\left[ \frac{\sinh \psi }{f_1}\left( C_0\,z\,f_3'\right) \right. \right. \nonumber \\&\quad \left. \left. +\frac{\cosh \psi }{f_2}\,\left( z\,f_3'+\lambda \,f_3\,\right) \right] \frac{2\,f_2\,\cosh \psi }{3} +\lambda \,f_3\right] \Bigg )\Bigg ] , \end{aligned}$$

160c $$\begin{aligned} g_3&= \frac{1}{f_1^2\,f_2^3\,f_3^2}\Bigg [-z^2\,f_1^2\,f_2\,f_3\,f_3''+z^2\,f_2\,f_3'^2\, \left( C_0^2\,f_2^2-f_1^2\right) +f_3'\,\Bigg (z^2\,f_3\,f_2'\,\left( 2\,C_0^2\,f_2^2+f_1^2\right) -4\,f_1\,f_2\,\nonumber \\&\quad \times \Bigg [\frac{z^2\,f_3\,f_1'}{4}+z\,\Bigg (-\bigg [\frac{\sinh \psi }{f_1}\left( C_0\,z\,f_3'\right) +\frac{\cosh \psi }{f_2}\,\left( z\,f_3'+\lambda \,f_3\,\right) \Bigg ]\frac{f_2}{4}\, \left( C_0\,f_2\,\sinh \psi +f_1\,\cosh \psi \right) \nonumber \\&\quad +f_1\,f_3\,\left( \lambda +\frac{1}{4}\right) \Bigg )\Bigg ]\Bigg ) -2\,f_1\,f_3 \Bigg (-\frac{z\,f_2'}{2}\Bigg (C_0\,f_2^2\,\sinh \psi \,\Bigg [\frac{\sinh \psi }{f_1}\left( C_0\,z\,f_3'\right) +\frac{\cosh \psi }{f_2}\,\left( z\,f_3'+\lambda \,f_3\,\right) \Bigg ]+\lambda \,f_1\,f_3\Bigg )\nonumber \\&\quad +f_2\,\Bigg [\frac{z\,f_1'}{2}\Bigg (-\Bigg [\frac{\sinh \psi }{f_1}\left( C_0\,z\,f_3'\right) +\frac{\cosh \psi }{f_2}\,\left( z\,f_3'+\lambda \,f_3\,\right) \Bigg ]\,f_2\,\cosh \psi +\lambda \,f_3\Bigg )\nonumber \\&\quad +\frac{z\,f_2\,\psi '}{2}\Bigg (-\left( C_0\,f_2\,\cosh \psi +f_1\,\sinh \psi \right) \, \left[ \frac{\sinh \psi }{f_1}\left( C_0\,z\,f_3'\right) +\frac{\cosh \psi }{f_2}\, \left( z\,f_3'+\lambda \,f_3\,\right) \right] +\lambda \,C_0\,f_3\Bigg )\nonumber \\&\quad -\frac{z\,f_2}{2}\sin \left( \arccos \left( -\left[ \frac{\sinh \psi }{f_1}\left( C_0\,z\,f_3'\right) +\frac{\cosh \psi }{f_2}\,\left( z\,f_3'+\lambda \,f_3\,\right) \right] \right) \right) \nonumber \\&\quad \times \left( \arccos \left( -\left[ \frac{\sinh \psi }{f_1}\left( C_0\,z\,f_3'\right) +\frac{\cosh \psi }{f_2}\,\left( z\,f_3'+\lambda \,f_3\,\right) \right] \right) \right) ' \left( C_0\,f_2\,\sinh \psi +f_1\,\cosh \psi \right) \nonumber \\&\quad +\lambda \,f_1\,\left( -\left[ \frac{\sinh \psi }{f_1}\left( C_0\,z\,f_3'\right) +\frac{\cosh \psi }{f_2}\,\left( z\,f_3'+\lambda \,f_3\,\right) \right] f_2\, \cosh \psi \,+\lambda \,f_3\right) \Bigg ]\Bigg )\Bigg ], \end{aligned}$$

160d $$\begin{aligned} g_4&= \frac{C_0}{f_1^2\,f_2^2\,f_3}\Bigg [-z^2\,f_1\,f_2\,f_3''+f_3'\,\left[ z^2\,\left( f_1\,f_2\right) '-z\, f_1\,f_2\right] +\lambda \,z\,f_1\,f_2'\,f_3\Bigg ], \end{aligned}$$

160e $$\begin{aligned} h_1&= \frac{1}{f_1^2\,f_2^2\,f_3} \Bigg [-\frac{f_3'\,z^2}{4}\,\left( C_0\,^2\,f_2^2+f_1^2\right) +\Bigg (-\frac{z^2\,f_1\,f_3\,f_1'}{4}+\frac{C_0^2\,z^2\,f_2\,f_3\,f_2'}{4}-\frac{z\,f_1}{2}\,\nonumber \\&\quad \times \Bigg [-\left[ \frac{\sinh \psi }{f_1}\left( C_0\,z\,f_3'\right) +\frac{\cosh \psi }{f_2}\, \left( z\,f_3'+\lambda \,f_3\,\right) \right] \frac{f_2}{2}\,\left( -C_0\,f_2\,\sinh \psi +f_1\,\cosh \psi \right) +\lambda \,f_1\,f_3\Bigg ]\Bigg )\Bigg ], \end{aligned}$$

160f $$\begin{aligned} h_2&=\frac{C_0}{f_1^2\,f_3} \Bigg [-\left[ \frac{\sinh \psi }{f_1}\left( C_0\,z\,f_3'\right) +\frac{\cosh \psi }{f_2}\,\left( z\,f_3'+\lambda \,f_3\,\right) \right] z\,f_1\,\sinh \psi -C_0\,z^2\,f_3'\Bigg ] , \end{aligned}$$

160g $$\begin{aligned} h_3&=-\frac{z}{2f_2^2\,f_3}\Bigg [z\,f_3'-\left[ \frac{\sinh \psi }{f_1}\left( C_0\,z\,f_3'\right) +\frac{\cosh \psi }{f_2}\,\left( z\,f_3'+\lambda \,f_3\,\right) \right] \, f_2\,\cosh \psi +\lambda \,f_3\Bigg ] , \end{aligned}$$

160h $$\begin{aligned} h_4&= \frac{1}{f_1\,f_2\,f_3}\Bigg [-z^2\,C_0\,f_3'-\frac{z}{2} \Bigg [-\left( C_0\,f_2\,\cosh \psi -f_1\,\sinh \psi \right) \,\left[ \frac{\sinh \psi }{f_1} \left( C_0\,z\,f_3'\right) +\frac{\cosh \psi }{f_2}\,\left( z\,f_3'+\lambda \,f_3\,\right) \right] +\lambda \,C_0\,f_3\Bigg ]\Bigg ] , \end{aligned}$$

160i $$\begin{aligned} m_1&= \frac{\lambda }{f_1\,f_2^2\,f_3} \Bigg [\frac{z\,f_3'\,f_1}{2} +\Bigg (\frac{z\,f_3\,f_1'+C_0\,z\,f_2\,f_3\,\psi '}{2} +f_1\,\left[ -\left[ \frac{\sinh \psi }{f_1} \left( C_0\,z\,f_3'\right) +\frac{\cosh \psi }{f_2}\,\left( z\,f_3'+\lambda \,f_3\,\right) \right] \frac{f_2\,\cosh \psi }{2}+\lambda \,f_3\right] \Bigg )\Bigg ] , \end{aligned}$$

160j $$\begin{aligned} m_2&=0 , \end{aligned}$$

160k $$\begin{aligned} m_3&=\frac{\lambda }{f_2^2\,f_3}\Bigg [z\,f_3'-\left[ \frac{\sinh \psi }{f_1}\left( C_0\,z\,f_3'\right) +\frac{\cosh \psi }{f_2}\,\left( z\,f_3'+\lambda \,f_3\,\right) \right] \, f_2\,\cosh \psi +\lambda \,f_3\Bigg ] , \end{aligned}$$

160l $$\begin{aligned} m_4&= \frac{1}{f_1\,f_2\,f_3}\Bigg [C_0\,\lambda \,z\,f_3'+\lambda \,f_1\,\sinh \psi \, \left[ \frac{\sinh \psi }{f_1}\left( C_0\,z\,f_3'\right) +\frac{\cosh \psi }{f_2}\, \left( z\,f_3'+\lambda \,f_3\,\right) \right] \Bigg ] . \end{aligned}$$

The $T_0(z)$ expression is the following:

161 $$\begin{aligned} T_0(z)&=\frac{1}{f_1^2\,f_2^2\,f_3^2}\Bigg [8\,f_1\,f_2\,\bigg [\frac{\sinh \psi }{f_1}\left( C_0\,z\,f_3'\right) \nonumber \\&\quad +\frac{\cosh \psi }{f_2}\,\left( z\,f_3'+\lambda \,f_3\,\right) \bigg ]\nonumber \\&\quad \times \Bigg (\left[ f_1\,\left( \lambda \,f_3+\frac{z\,f_3'}{2}\right) +\frac{z\,f_3 \left( \psi '\,C_0\,f_2+f_1'\right) }{2}\right] \cosh \psi \nonumber \\&\quad +\frac{z\,\sinh \psi }{2}\left[ f_1\,f_3\,\psi '+C_0\, \left( f_2\,f_3\right) '\right] \Bigg ) +4\,z\,\sin \nonumber \\&\quad \times \left( \arccos \left( -\left[ \frac{\sinh \psi }{f_1}\left( C_0\,z\,f_3'\right) +\frac{\cosh \psi }{f_2}\,\left( z\,f_3'+\lambda \,f_3\,\right) \right] \right) \right) \cosh \nonumber \\&\quad \times \psi \,\left( \!\arccos \left( \!-\left[ \frac{\sinh \psi }{f_1}\left( C_0\,z\,f_3'\right) +\frac{\cosh \psi }{f_2}\, \left( z\,f_3'+\lambda \,f_3\,\right) \right] \right) \right) '\nonumber \\&\quad \times f_1^2\,f_2\,f_3+4\,z\,\sinh \psi \,\sin \bigg (\arccos \bigg (-\bigg [\frac{\sinh \psi }{f_1}\left( C_0\,z\,f_3'\right) \nonumber \\&\quad +\frac{\cosh \psi }{f_2}\,\left( z\,f_3'+\lambda \,f_3\,\right) \bigg ]\bigg )\bigg )\,\nonumber \\&\quad \times \left( \arccos \left( -\left[ \frac{\sinh \psi }{f_1}\left( C_0\,z\,f_3'\right) +\frac{\cosh \psi }{f_2}\, \left( z\,f_3'+\lambda \,f_3\,\right) \right] \right) \right) '\nonumber \\&\quad \times C_0\,f_1\,f_2^2\,f_3\nonumber \\&\quad +f_1^2\,\big [-6\lambda ^2\,f_3^2-8\lambda \,z\,f_3\,f_3'-2\,z^2\,f_3'^2-2\,f_2^2\big ]\nonumber \\&\quad -4\,f_1\,f_3\left[ \lambda \,C_0\,z\,f_2\,f_3\,\psi '+f_1'\left( \lambda \,z\,f_3+z^2\,f_3'\right) \right] \nonumber \\&\quad +2\,C_0^2\,z^2\,f_2\,f_3'\left( f_2\,f_3'+2\,f_3\,f_2'\right) \Bigg ]. \end{aligned}$$

## Code availability

This manuscript has no associated code/software. [Authors’ comment: Code/Software sharing not applicable to this article as no code/software was generated or analysed during the current study.]
